# Supplementary material for: BaTwa populations from Zambia retain ancestry of past hunter-gatherer groups
Source: Nat Commun. 2024 Aug 24;15:7307. doi: 10.1038/s41467-024-50733-y (PMC11344834; doi:10.1038/s41467-024-50733-y)
Supplement: Supplementary file 1 — Supplementary Information [file 41467_2024_50733_MOESM1_ESM.pdf]

BaTwa populations from Zambia retain ancestry of past hunter-gatherer groups

Gwenna Breton, Lawrence Barham, George Mudenda,
Himla Soodyall, Carina M. Schlebusch, Mattias Jakobsson

Supplementary Figures

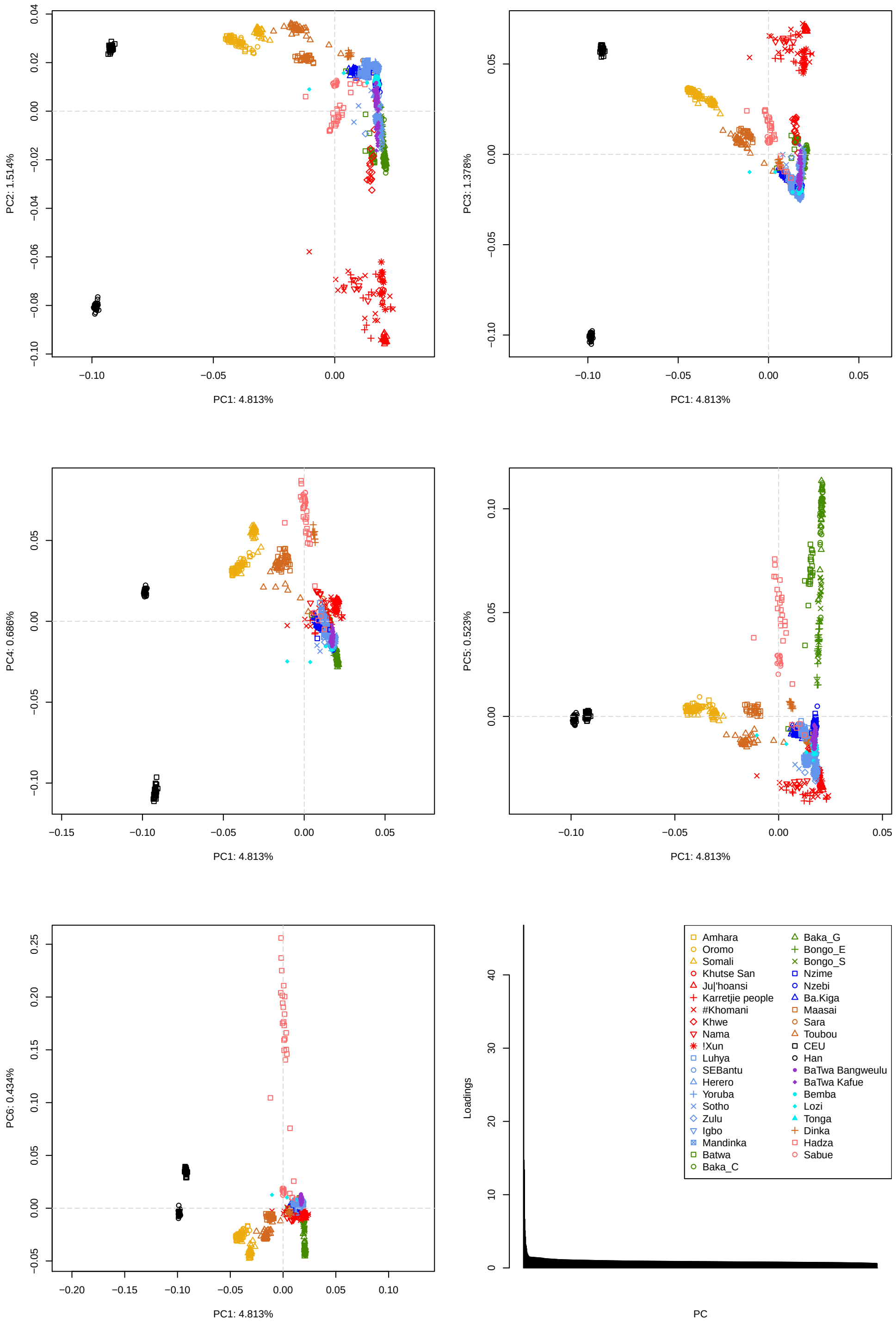

Fig. S1 PCA of the Omni1 dataset (first six PC axes).

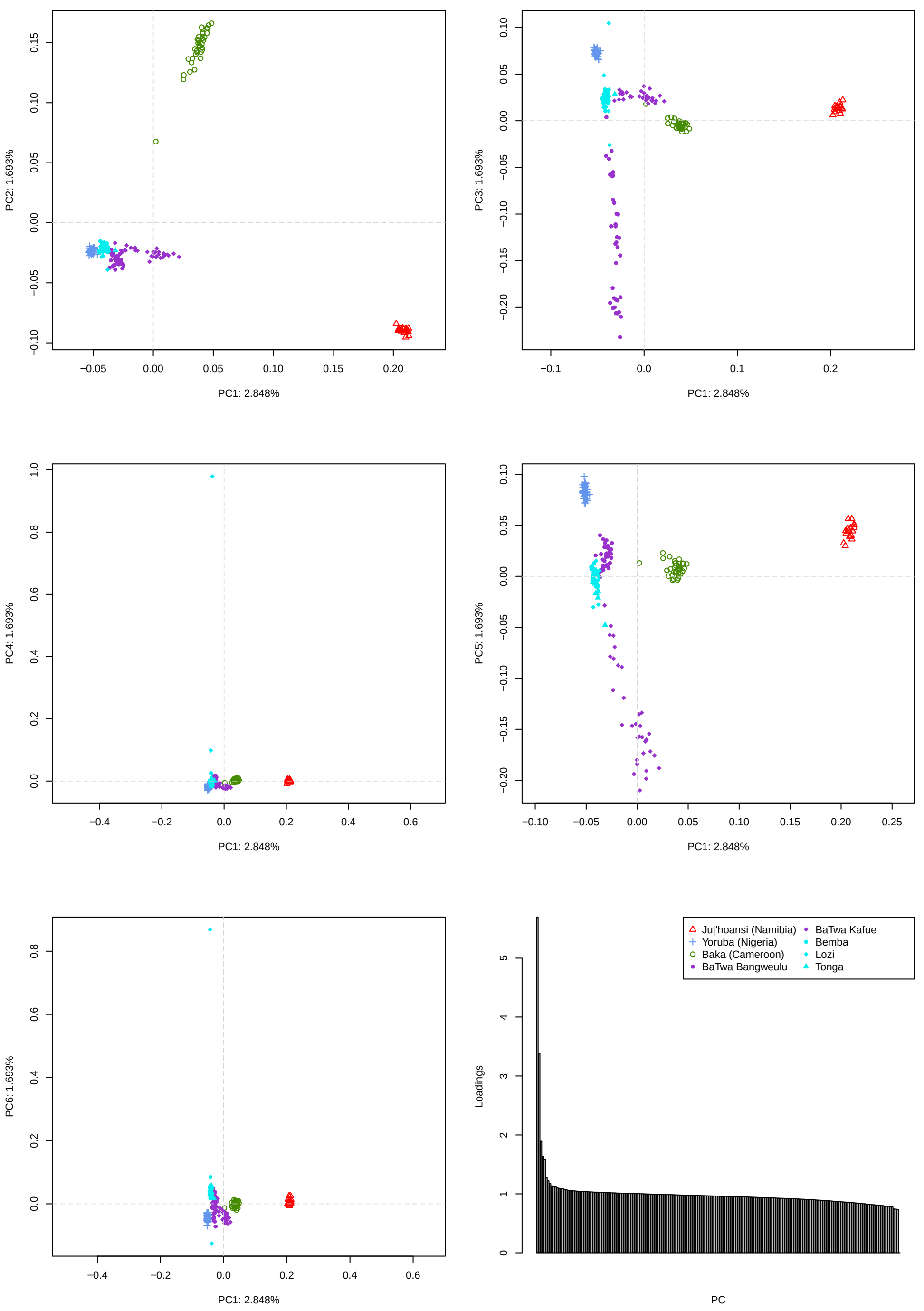

Fig. S2 PCA of a subset of the Omni1 dataset: the five Zambian populations, Yoruba, Ju|'hoansi and Baka (Cameroon).

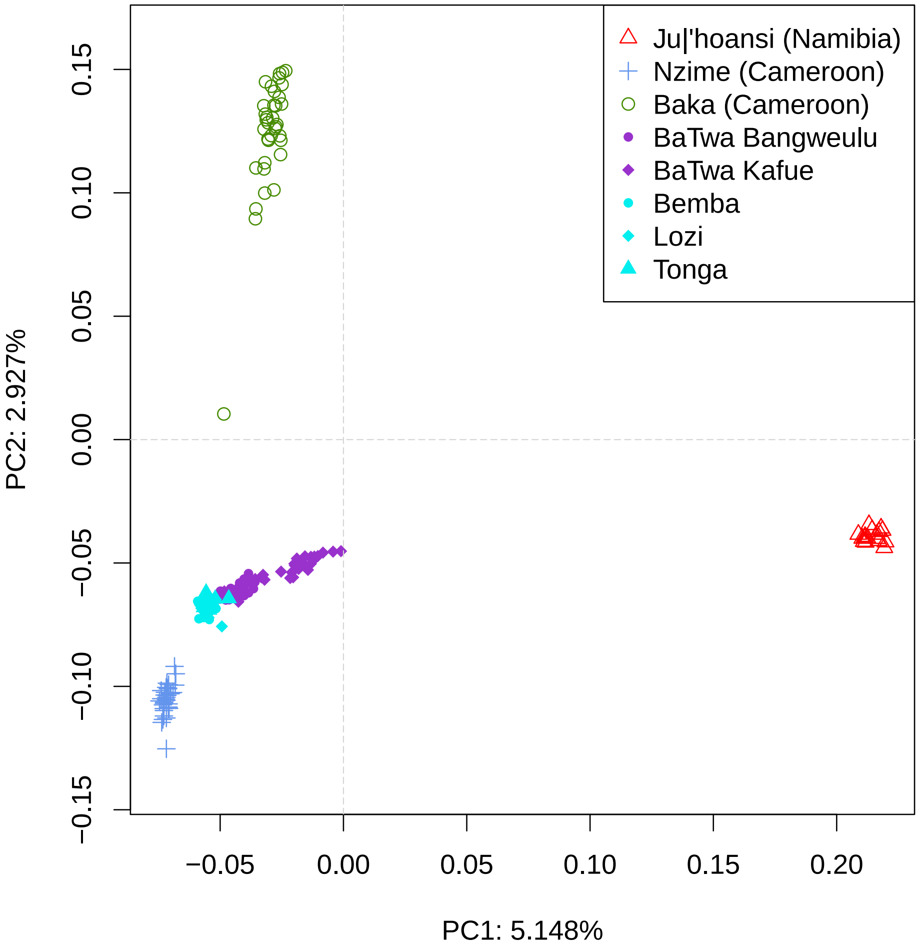

Fig. S3 PCA (first two axes) of a subset of the Omni1 dataset (Nzime, Ju|'hoansi and Baka (Cameroon)) and projection of the five Zambian populations.

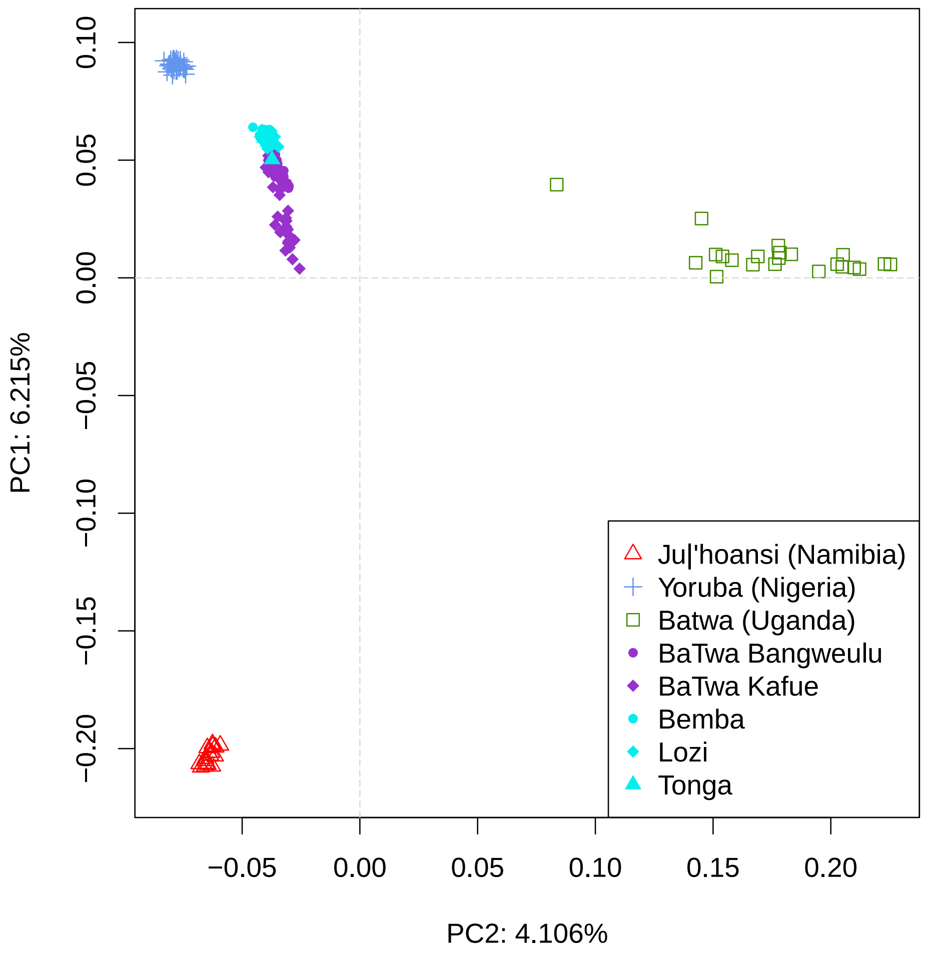

Fig. S4 PCA (first two axes) of a subset of the Omni1 dataset (Yoruba, Ju|'hoansi and BaTwa from Uganda) and projection of the five Zambian populations. Figure 1c is the same analysis but with the Baka instead of the BaTwa.

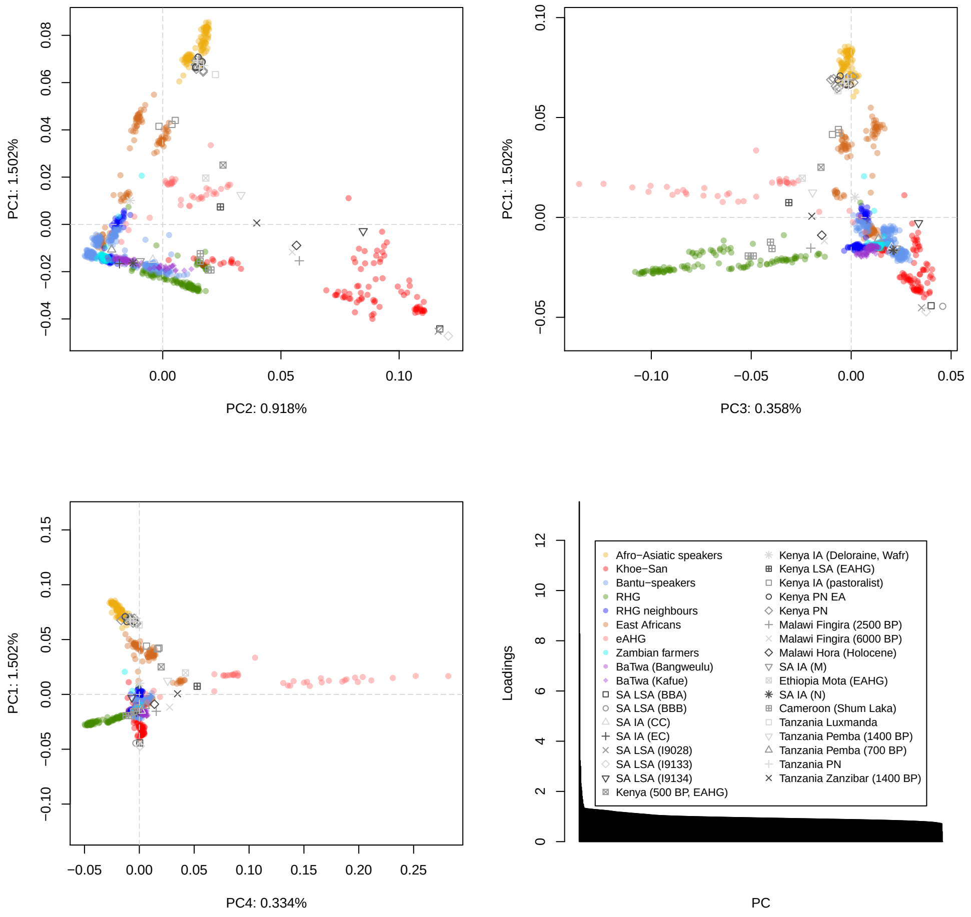

Fig. S5 PCA including ancient samples. Present-day samples are represented by filled colored disks, while ancient samples are represented by gray symbols. SA: South Africa, LSA: Late Stone Age, IA: Iron Age, PN: Pastoral Neolithic.

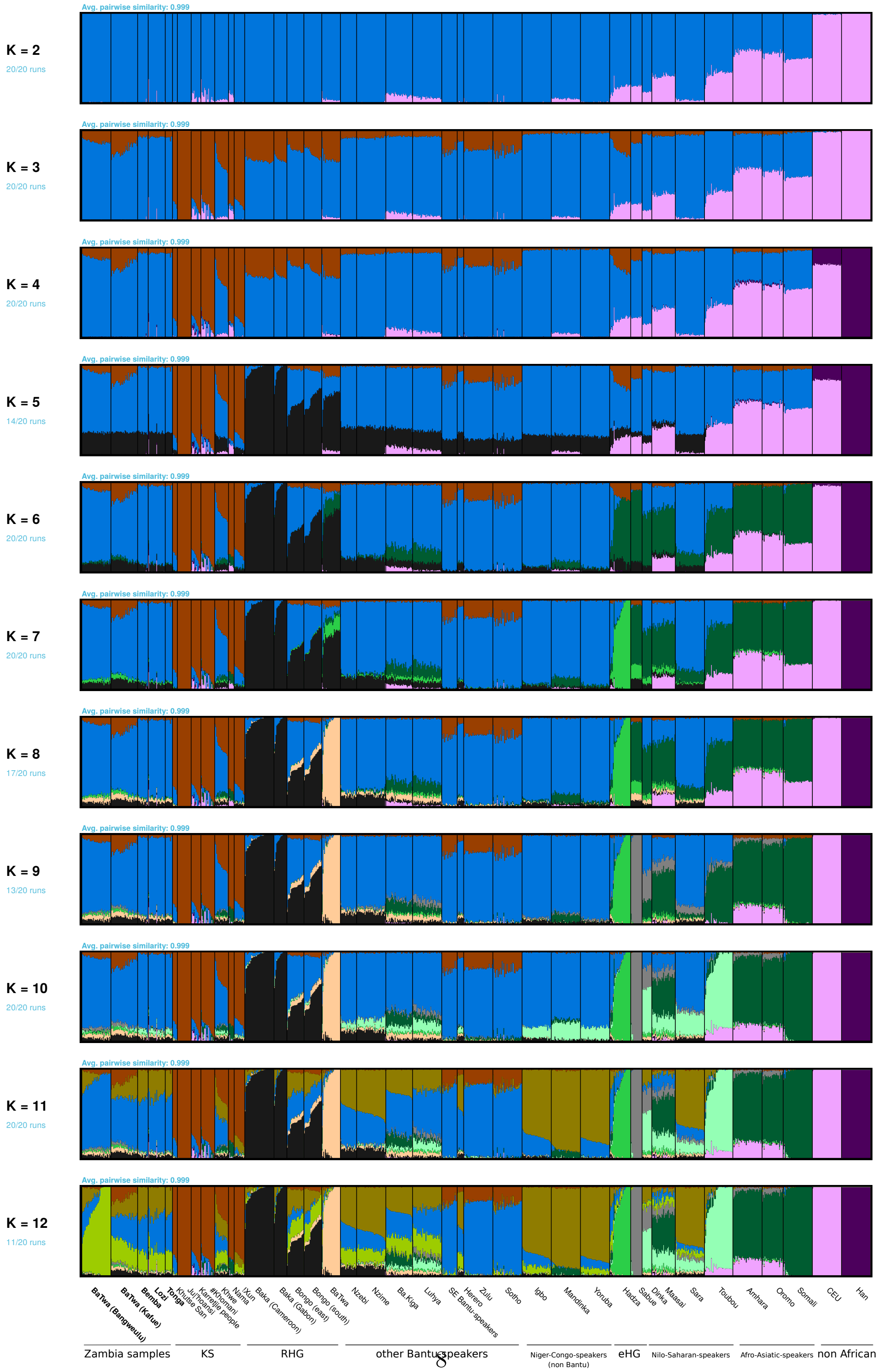

Fig. S6 Unsupervised clustering results (ADMIXTURE) of the Omni1 dataset, from two to twelve putative clusters.

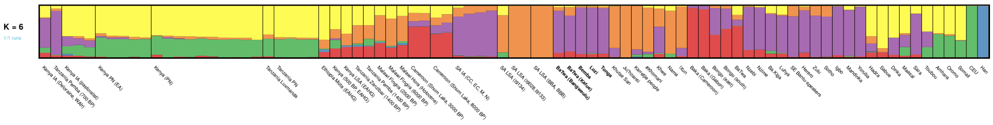

Fig. S7 Unsupervised clustering results (ADMIXTURE) of the Omni1 dataset combined with ancient samples showing the representative run for six putative clusters. For the present-day populations, the population average is shown and the width of the bar is not representative of sample size. See Supplementary Figure S8 for the complete results.

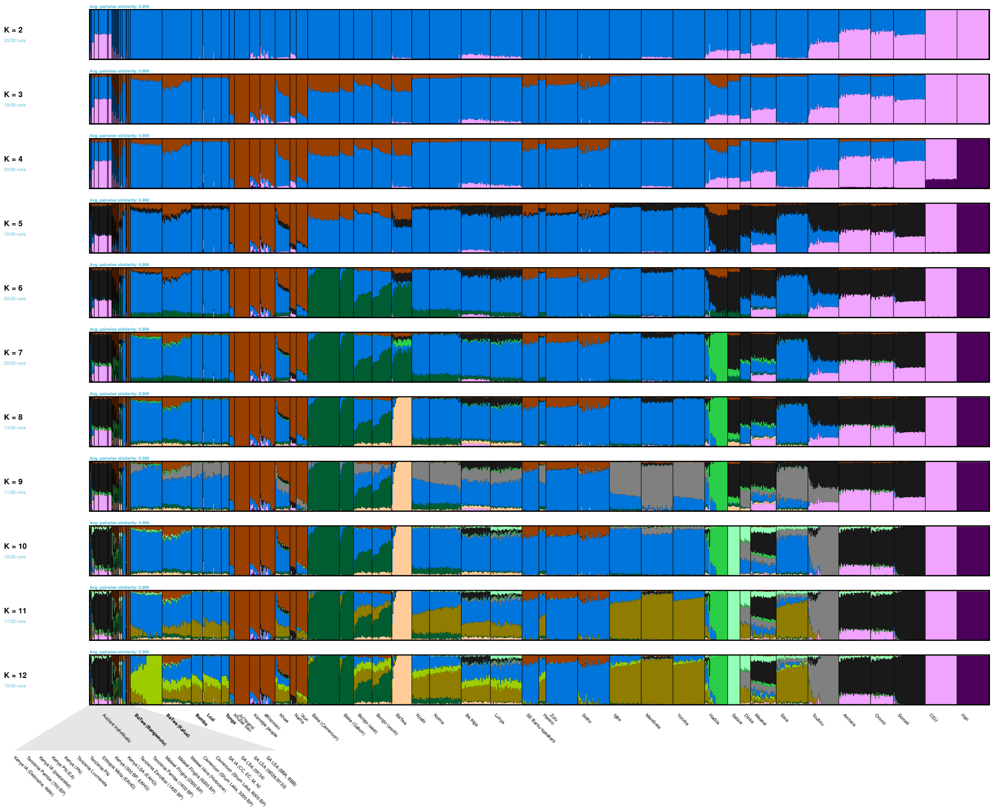

Fig. S8 Unsupervised clustering results (ADMIXTURE) of the Omni1 dataset combined with ancient samples, from two to twelve putative clusters. See Supplementary Fig. S7 for a closer view of the ancient samples (on the left).

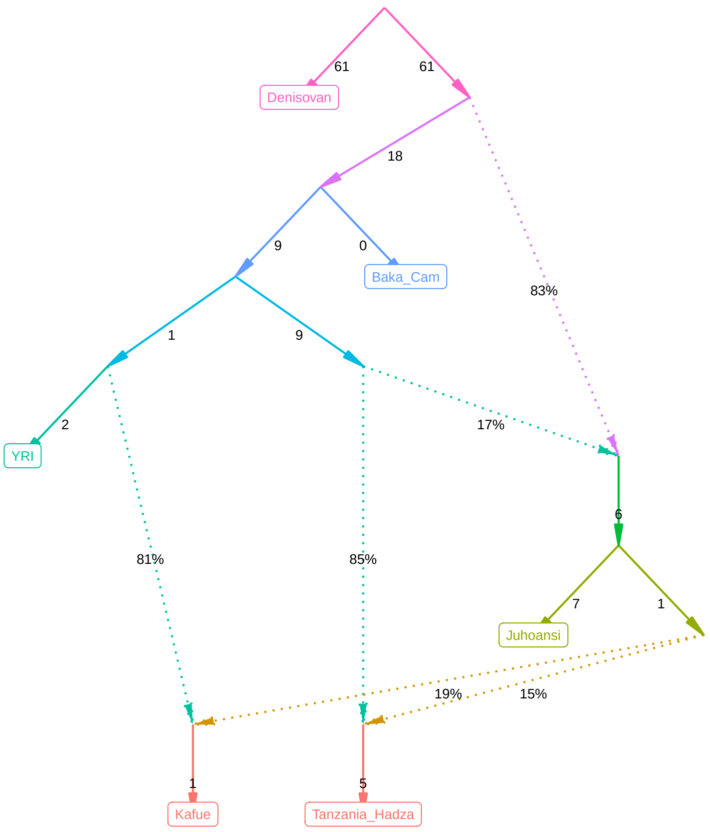

Fig. S9 Admixture graph with the lowest likelihood for the BaTwa from Kafue, assuming three admixture events for the specific set of populations presented in the figure. YRI: Yoruba.

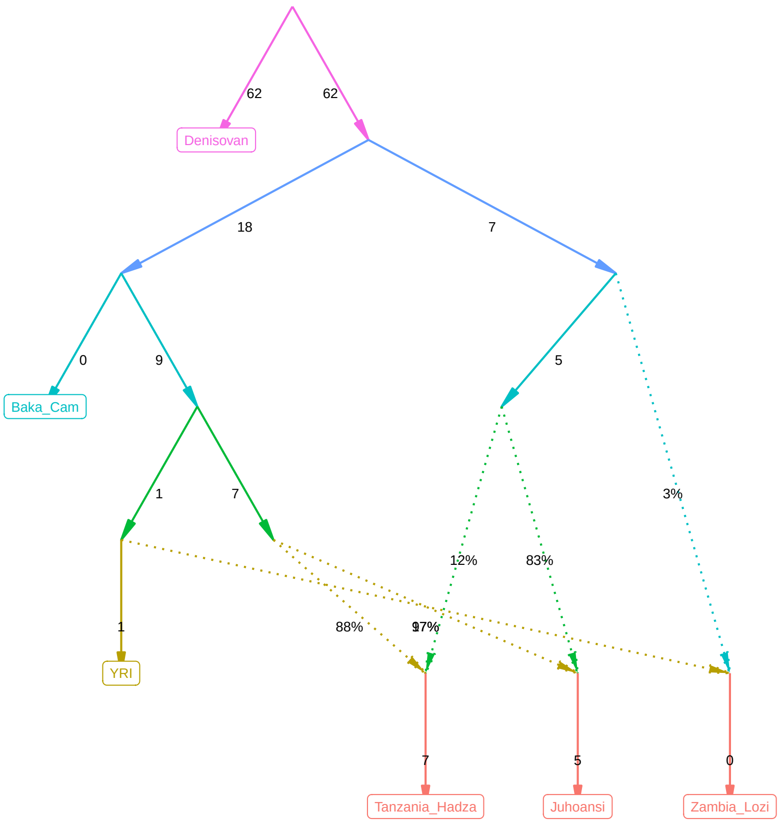

Fig. S10 Admixture graph with the lowest likelihood for the Lozi, assuming three admixture events for the specific set of populations presented in the figure. Two Lozi individuals with recent non-African admixture were excluded from the analyses. YRI: Yoruba.

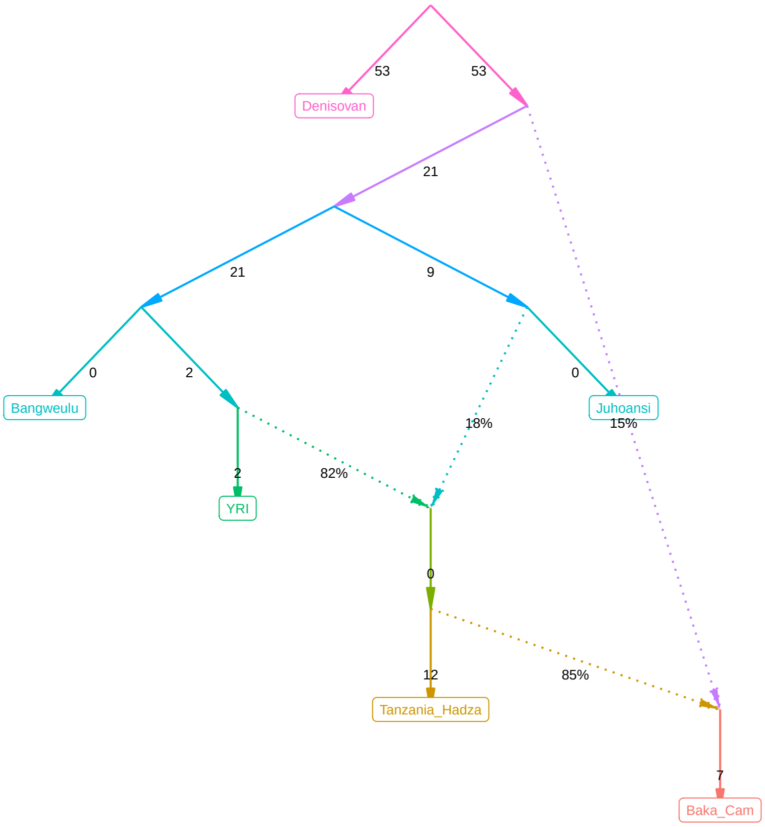

Fig. S11 Admixture graph with the lowest likelihood for the BaTwa from Bangweulu, assuming two admixture events for the specific set of populations presented in the figure. YRI: Yoruba.

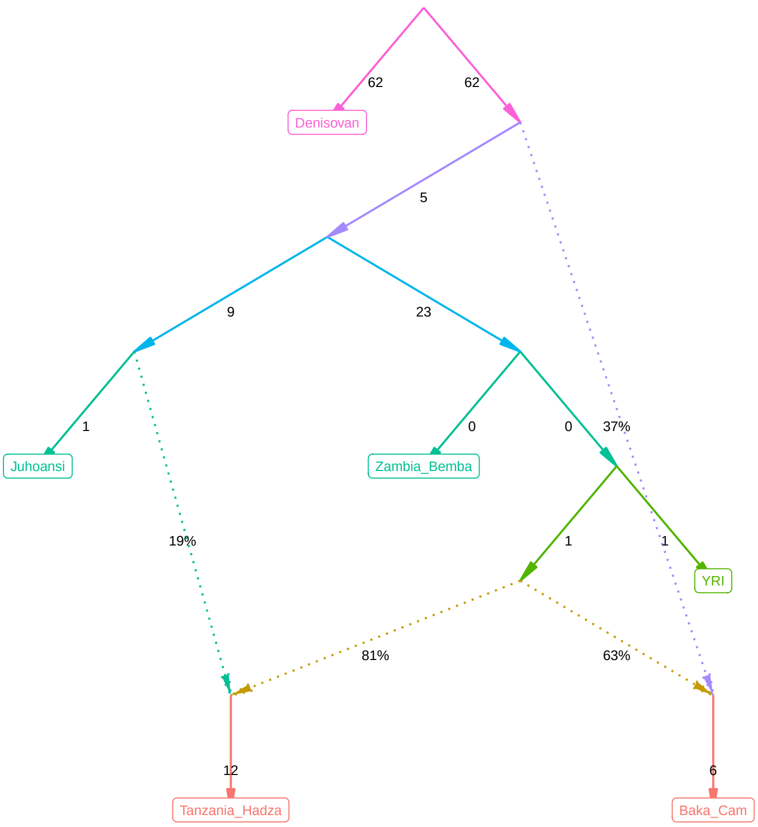

Fig. S12 Admixture graph with the lowest likelihood for the Bemba, assuming two admixture events for the specific set of populations presented in the figure. YRI: Yoruba.

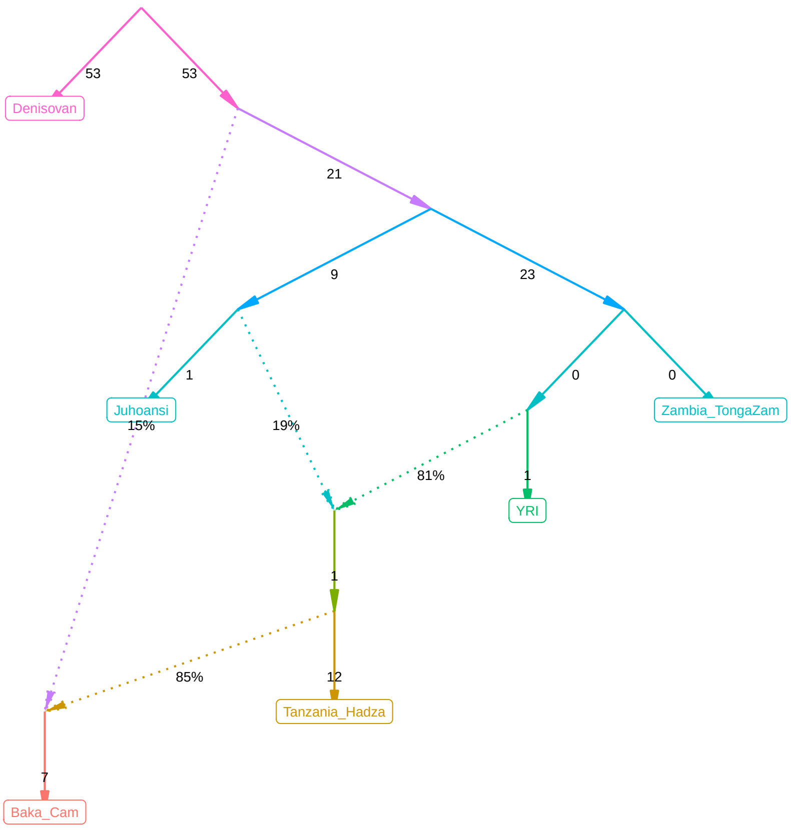

Fig. S13 Admixture graph with the lowest likelihood for the Tonga, assuming two admixture events for the specific set of populations presented in the figure. YRI: Yoruba.

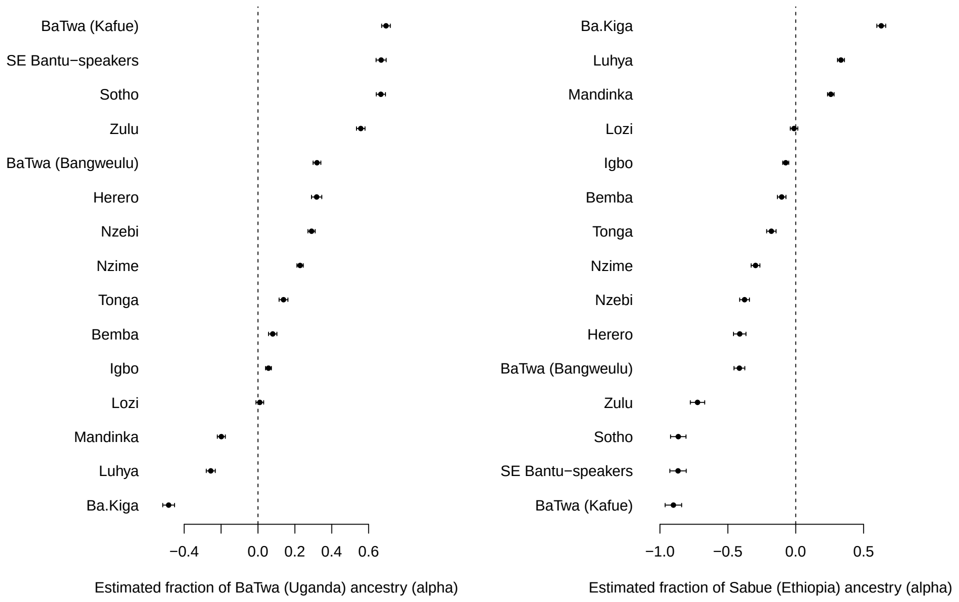

Fig. S14 Admixture proportions (α) estimated with f_4 ratio test. The test is of the form: f_4(Han Chinese, ancestral; target, Yoruba)/f_4(Han Chinese, ancestral; admixture source, Yoruba). The target populations are listed on the left. The bars represent two standard deviations. 337,051 SNP and 547 blocks for the block jacknife were used for the computation. **Left panel:** Admixture source: eastern rainforest hunter-gatherer (BaTwa from Uganda). **Right panel:** Admixture source: eastern African hunter-gatherer (Sabue). Similar analyses were performed for more admixture sources: Khoe-San, western rainforest hunter-gatherer, and another eastern African hunter-gatherer (Figure 3, Supplementary Fig. S15). The results between the two rainforest hunter-gatherer ancestries -Baka and BaTwa-, and between the two eastern African hunter-gatherer ancestries -Hadza and Sabue-, are strongly correlated (Spearman rho correlation, two-sided, p-value¡2.2×10^{-16}, rho=1 (positive correlation).

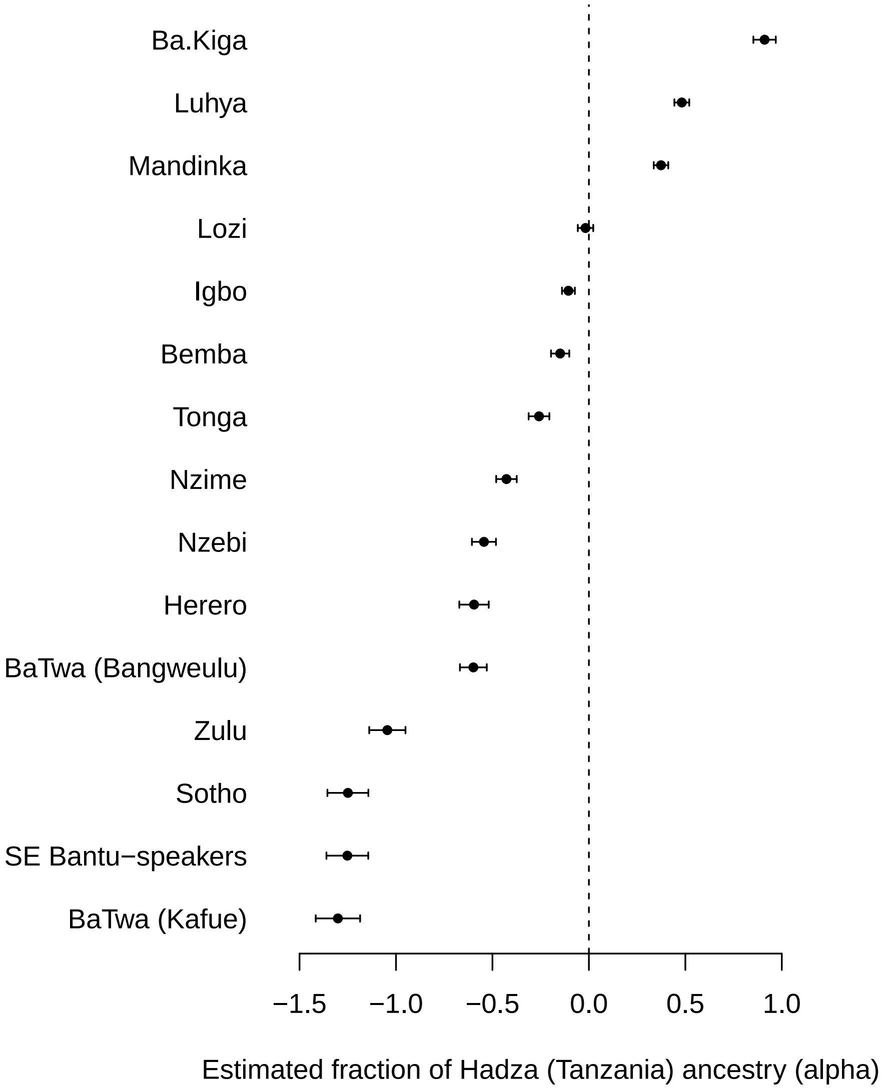

Fig. S15 Admixture proportions (α) estimated with f_4 ratio for the admixture source eastern African hunter-gatherer (Hadza). The target populations are listed on the left. The bars represent two standard deviations. 337,051 SNP and 547 blocks for the block jacknife were used for the computation.

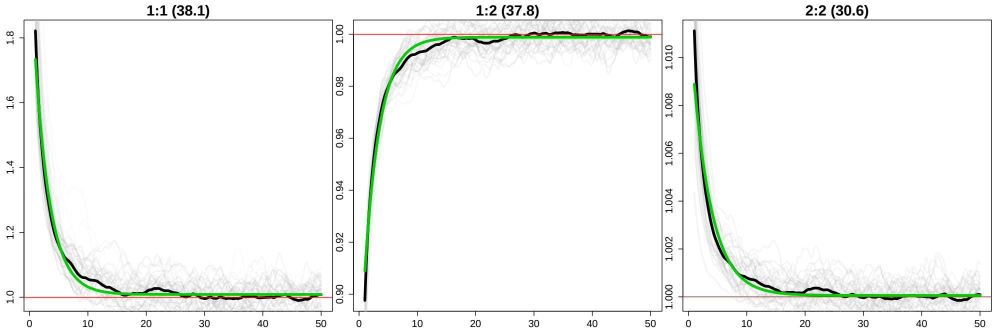

Fig. S16 Co-ancestry curves for two-way admixture in the BaTwa from Bangweulu. x-axis: genetic distance (in centimorgans). y-axis: exponential decay of the ratio of probabilities of pairs of local ancestries. Black lines, empirical coancestry curves across all target individuals. Light grey lines, empirical coancestry curves per individual. Green line, fitted single-event coancestry curve. The middle panel is the co-ancestry of the minor and major ancestries. The number in bracket is the estimated admixture time in generations.

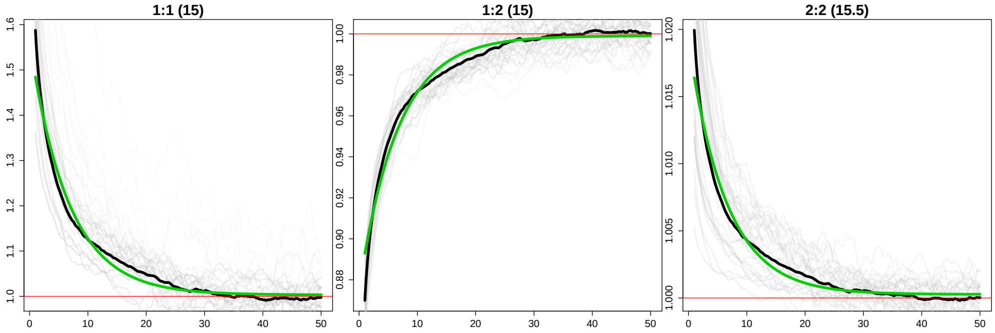

Fig. S17 Co-ancestry curves for two-way admixture in the BaTwa from Kafue. x-axis: genetic distance (in centimorgans). y-axis: exponential decay of the ratio of probabilities of pairs of local ancestries. Black lines, empirical coancestry curves across all target individuals. Light grey lines, empirical coancestry curves per individual. Green line, fitted single-event coancestry curve. The middle panel is the co-ancestry of the minor and major ancestries. The number in bracket is the estimated admixture time in generations.

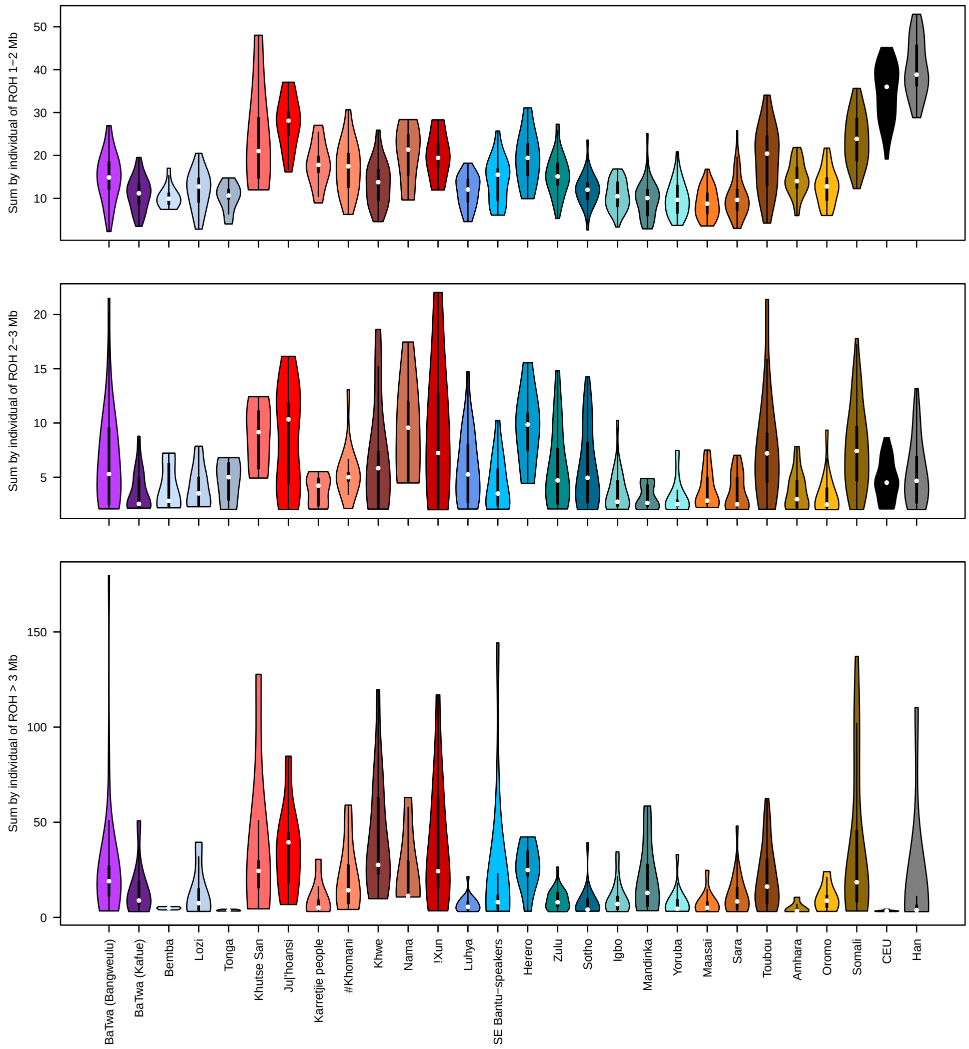

Fig. S18 Total runs of homozygosity length by individual by population, subdivided in three length classes. **Top:** class 1-2 Mb. Sample size left to right: 36, 33, 13, 21, 9, 6, 17, 12, 17, 17, 7, 13, 36, 19, 8, 36, 36, 36, 36, 36, 29, 36, 35, 36, 26, 36, 36, 36. **Middle:** class 2-3 Mb. Sample size left to right: 32, 22, 10, 18, 6, 6, 17, 10, 15, 16, 7, 13, 27, 16, 7, 33, 34, 22, 16, 18, 21, 17, 30, 24, 14, 35, 32, 33. **Bottom:** class > 3 Mb. Sample size left to right: 32, 20, 3, 11, 3, 6, 17, 6, 12, 15, 6, 13, 19, 14, 8, 29, 20, 20, 15, 14, 9, 20, 32, 6, 6, 34, 7, 9.

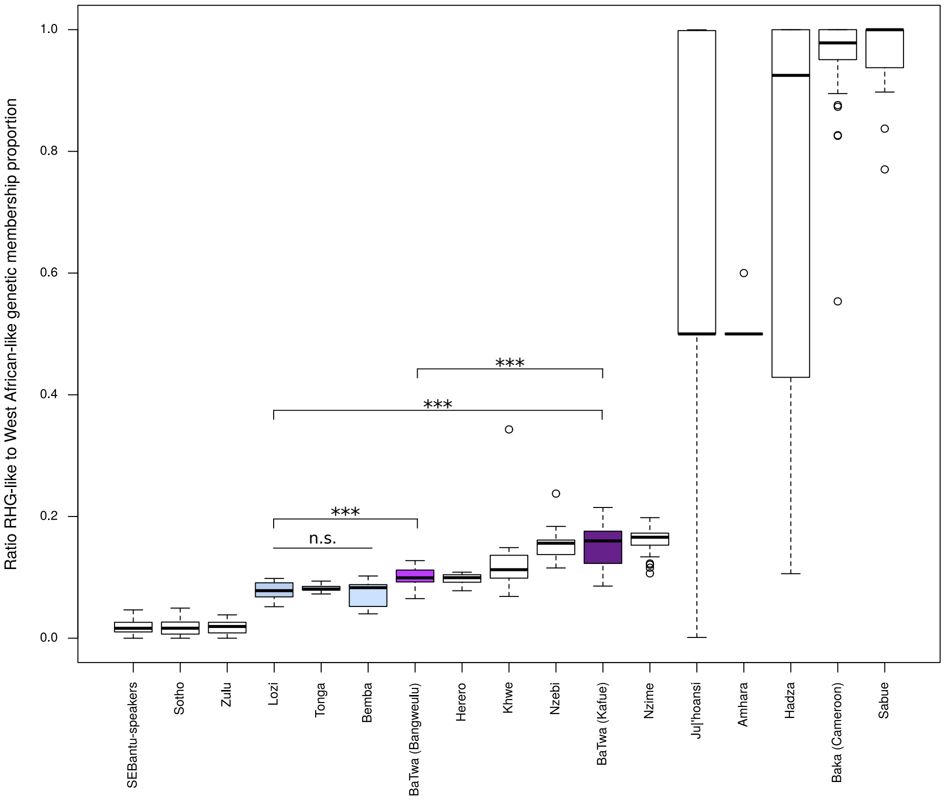

Fig. S19 Ratio of the rainforest-hunter-gatherer-like to the western-African-like genetic membership as estimated with ADMIXTURE with six putative clusters. Center line, median; box limits, lower and upper quartiles; whiskers, 1.5x interquartile range or minimal/maximal value; circles, outliers. Some one-tailed t-tests results were added; ***: p-value≤0.001, n.s.: non significant. Exact p-values: Lozi-BaTwa (Bangweulu): 6.336e-07; Lozi-BaTwa (Kafue): 1.471e-14; BaTwa (Bangweulu)-BaTwa (Kafue): 3.836e-10.

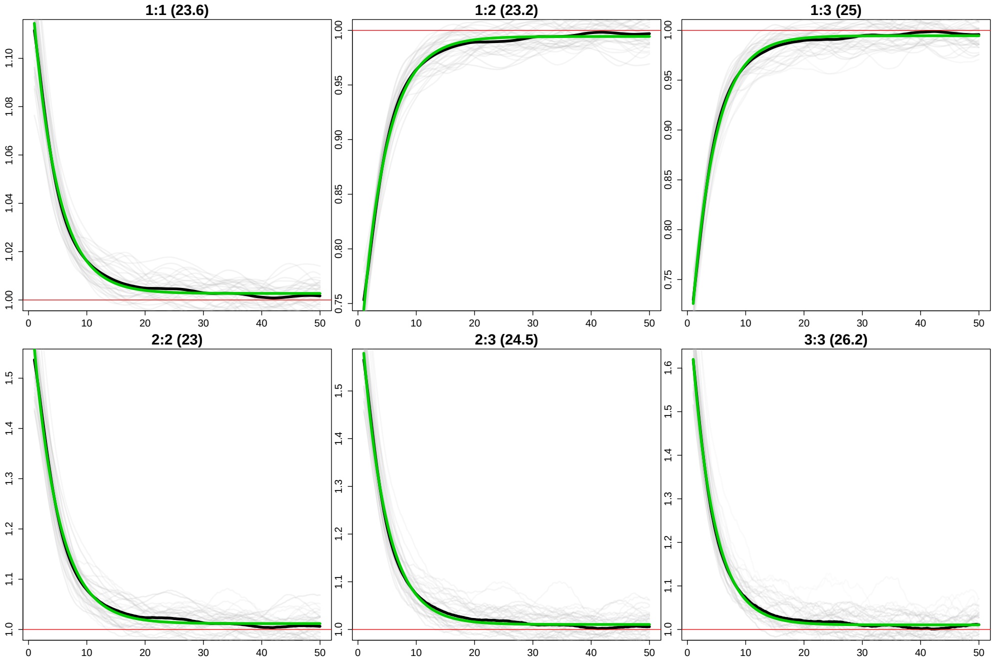

Fig. S20 Co-ancestry curves for three-way admixture in the BaTwa from Bangweulu, source populations specified: Yoruba, Baka (Cameroon), Ju|'hoansi. x-axis: genetic distance (in centimorgan). y-axis: exponential decay of the ratio of probabilities of pairs of local ancestries. Black lines, empirical coancestry curves across all target individuals. Light grey lines, empirical coancestry curves per individual. Green line, fitted single-event coancestry curve. Top middle panel, co-ancestry of Yoruba and Baka. Top right panel, co-ancestry of Yoruba and Ju|'hoansi. Bottom middle panel, co-ancestry of Baka and Ju|'hoansi. The numbers in bracket are the estimated admixture times in generations.

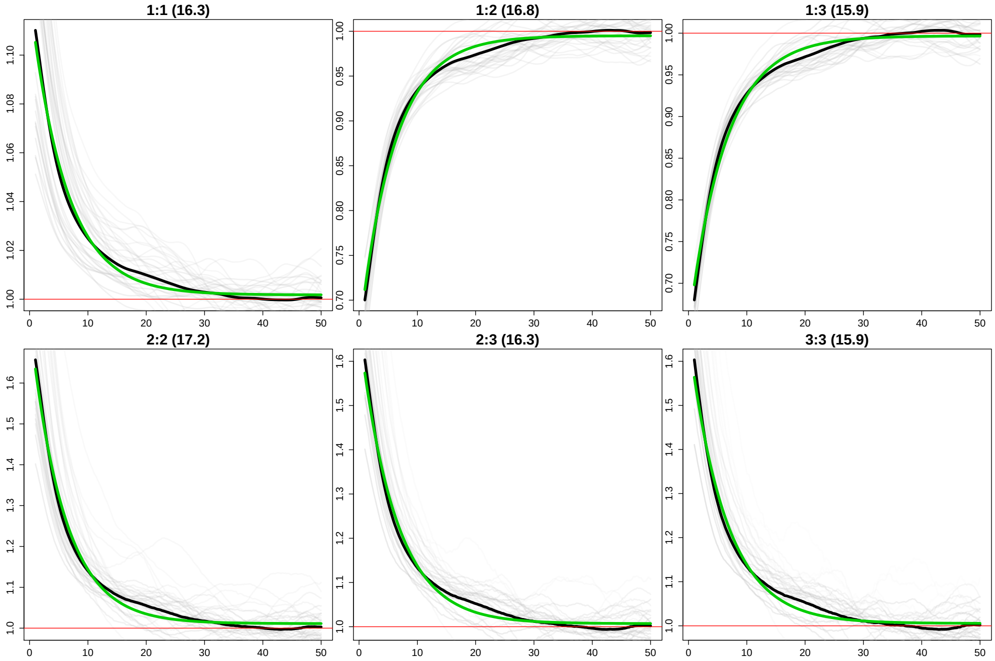

Fig. S21 Co-ancestry curves for three-way admixture in the BaTwa from Kafue, source populations specified: Yoruba, Baka (Cameroon), Ju|'hoansi. x-axis: genetic distance (in centimorgans). y-axis: exponential decay of the ratio of probabilities of pairs of local ancestries. Black lines, empirical coancestry curves across all target individuals. Light grey lines, empirical coancestry curves per individual. Green line, fitted single-event coancestry curve. Top middle panel, co-ancestry of Yoruba and Baka. Top right panel, co-ancestry of Yoruba and Ju|'hoansi. Bottom middle panel, co-ancestry of Baka and Ju|'hoansi. The numbers in bracket are the estimated admixture times in generations.

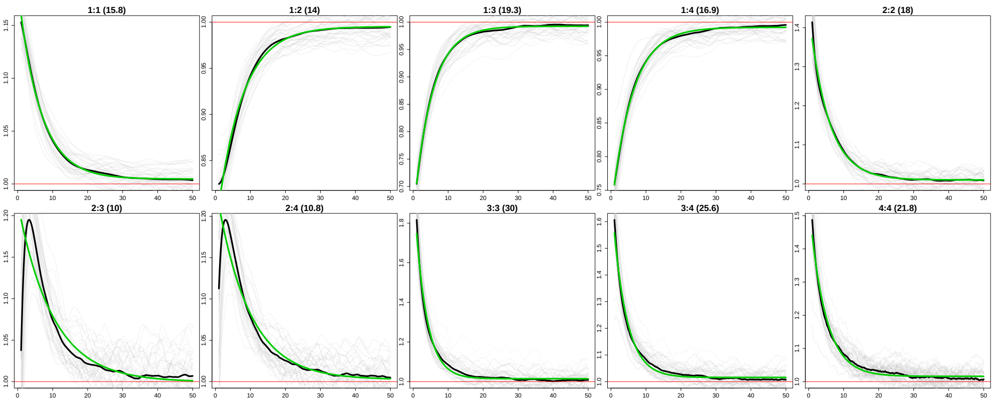

Fig. S22 Co-ancestry curves for four-way admixture in the BaTwa from Bangweulu, source populations specified: Yoruba, Baka (Cameroon), Ju|'hoansi, Amhara. x-axis: genetic distance (in centimorgans). y-axis: exponential decay of the ratio of probabilities of pairs of local ancestries. Black lines, empirical coancestry curves across all target individuals. Light grey lines, empirical coancestry curves per individual. Green line, fitted single-event coancestry curve. Top second panel from the left, co-ancestry of Yoruba and Baka. Top third panel from the left, co-ancestry of Yoruba and Ju|'hoansi. Top fourth panel from the left, co-ancestry of Yoruba and Amhara. The numbers in bracket are the estimated admixture times in generations.

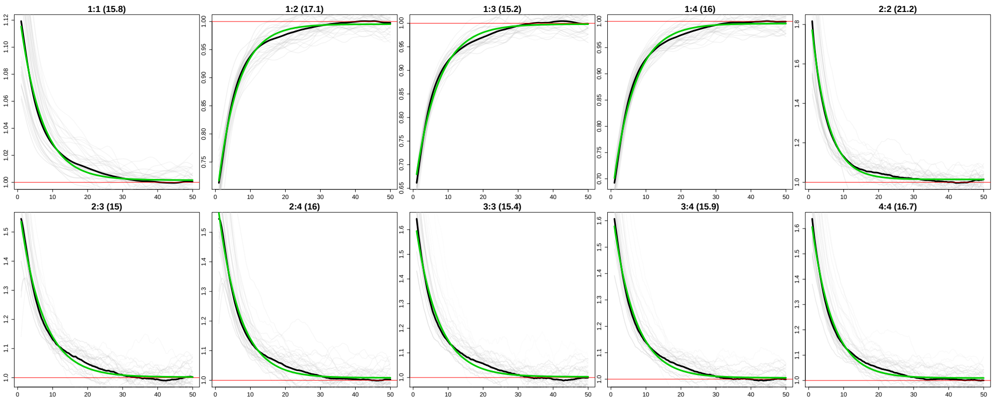

Fig. S23 Co-ancestry curves for four-way admixture in the BaTwa from Kafue, source populations specified: Ju|'hoansi, Baka (Cameroon), Yoruba, Amhara. x-axis: genetic distance (in centimorgans). y-axis: exponential decay of the ratio of probabilities of pairs of local ancestries. Black lines, empirical coancestry curves across all target individuals. Light grey lines, empirical coancestry curves per individual. Green line, fitted single-event coancestry curve. Top second panel from the left, co-ancestry of Yoruba and Baka. Top third panel from the left, co-ancestry of Yoruba and Ju|'hoansi. Top fourth panel from the left, co-ancestry of Yoruba and Amhara. The numbers in bracket are the estimated admixture times in generations.

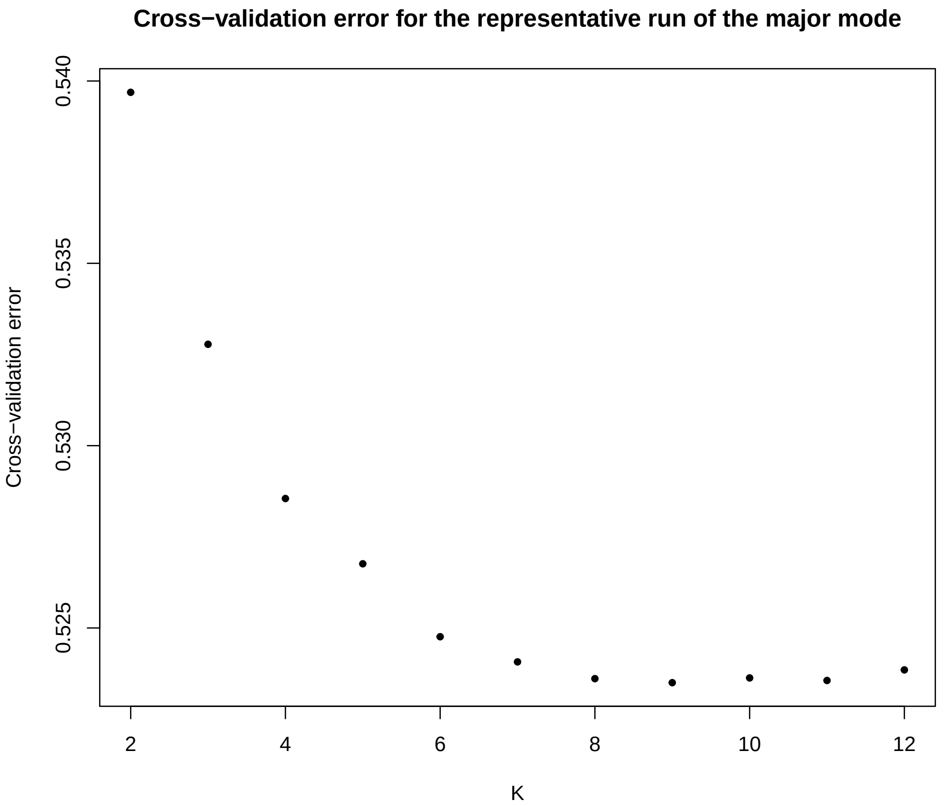

Fig. S24 Cross-validation error for the representative run of the major mode in the ADMIXTURE analysis. Y-axis: K, the number of putative genetic clusters. The major run was obtained with pong [1]. The errors correspond to ADMIXTURE runs independent from those shown in Supplementary Fig. S6.

Supplementary Tables

Population	n[1]	n_{final}[2]	Dataset	Language family	Array	Country of origin	Language – detail
Amhara	42	36	[2]	AfroAsiatic	Omni2.5	Ethiopia	Semitic
Ba.Kiga	40	33	[3]	NigerCongo	Omni1	Uganda	J.10, Bantu (Central)
Baka (Cameroon)	83	36	[3]	NigerCongo	Omni1	Cameroon	Baka-Gundi, Bantu
Baka (Gabon)	20	16	[3]	NigerCongo	Omni1	Gabon	Baka-Gundi, Bantu
BaTwa (Bangweulu)	40	36	new	NigerCongo	H3Africa	Zambia	Dialect of Bemba, Bantu
BaTwa (Kafue)	40	33	new	NigerCongo	H3Africa	Zambia	Twa (dialect of Tonga or Ila), Bantu
BaTwa	40	23	[3]	NigerCongo	Omni1	Uganda	J.10, Bantu (Central)
Bemba	13	13	[4]	NigerCongo	H3Africa	Zambia	Bemba, Bantu
Bongo (east)	24	21	[3]	NigerCongo	Omni1	Gabon (east)	B.60-70, Bantu (Northwest)
Bongo (south)	25	22	[3]	NigerCongo	Omni1	Gabon (south)	B.30, Bantu (Northwest)
CEU	60	36	[5]	IndoEuropean	Omni2.5	US	Indo-European
Dinka	12	12	[6]	NiloSaharan	Omni1	Sudan	Dinka
Hadza	26	26	[6]	Khoisan	Omni1	Tanzania	Hadza
Han Chinese (CHB)	60	36	[5]	SinoTibetan	Omni2.5	China	Sino-Tibetan
Igbo	99	36	[2]	NigerCongo	Omni2.5	Nigeria	Igboid
Ju|'hoansi	17	17	[7]	Khoisan	Omni2.5	Namibia	Southeast, Khoisan-Ju
Karretjie people	12	12	[7]	Khoisan	Omni2.5	South Africa	!Ui and KhoeKhoe, Khoisan-Tuu and Khoisan-Khoe
#Khomani	17	17	[7]	Khoisan	Omni2.5	South Africa	Taa and KhoeKhoe, Khoisan-Tuu and Khoisan Khoe
Khutse San	7	6	[7]	Khoisan	Omni2.5	Botswana	Kalahari, Khoisan-Khoe
Khwe	17	17	[7]	Khoisan	Omni2.5	Angola	Kalahari, Khoisan-Khoe
Lozi	21	21	[4]	NigerCongo	H3Africa	Zambia	Lozi, Bantu
Luyha (LWK)	60	36	[5]	NigerCongo	Omni2.5	Kenya	Luhya, Bantu
Maasai (MKK)	60	29	[5]	NiloSaharan	Omni2.5	Kenya	Maa/Maasai
Mandinka	88	36	[2]	NigerCongo	Omni2.5	Gambia	Mande
Nama	7	7	[7]	Khoisan	Omni2.5	Namibia	Nama, KhoeKhoe
Nzebi	20	20	[3]	NigerCongo	Omni1	Gabon	B.50, Bantu (Northwest)
Nzime	75	36	[3]	NigerCongo	Omni1	Cameroon	A.80, Bantu (Northwest)
Oromo	26	26	[2]	AfroAsiatic	Omni2.5	Ethiopia	Cushitic
Sabue	14	14	[6]	NiloSaharan	Omni1	Ethiopia	Sabue
Sara	62	36	[8]	NiloSaharan	Omni2.5	Chad	NA
Southeastern Bantu-speakers (SEBantu)	19	19	[7]	NigerCongo	Omni2.5	South Africa	Bantu (Zulu, Sotho, Tswana)
Somali	39	36	[2]	AfroAsiatic	Omni2.5	Ethiopia	Cushitic
Sotho	86	36	[2]	NigerCongo	Omni2.5	South Africa	Sotho, Bantu
Herero (SWBantu)	8	8	[7]	NigerCongo	Omni2.5	Namibia	Herero, Bantu
Tonga	10	9	[4]	NigerCongo	H3Africa	Zambia	Tonga, Bantu
Toubou	73	35	[8]	NiloSaharan	Omni2.5	Chad	NA
!Xun	13	13	[7]	Khoisan	Omni2.5	Angola	Northwest, Khoisan-Ju
Yoruba (YRI)	60	36	[5]	NigerCongo	Omni2.5	Nigeria	Yoruba, Yoruboid
Zulu	100	36	[2]	NigerCongo	Omni2.5	South Africa	Zulu, Bantu
Total	1535	973					

Table S1 Information about the populations (present-day samples).

[1]Sample size.

[2]Sample size after quality and relatedness filtering and downsampling.

Population	KS-like	RHG-like	eHG-like	KS + RHG + eHG
BaTwa (Bangweulu)	39.81 (25.07-50.15)	48.52 (37.01-65.00)	11.67 (0.01-18.03)	18.93 (0.48)
BaTwa (Kafue)	50.47 (37.32-61.14)	39.72 (30.06-62.68)	9.81 (0.01-16.04)	31.29 (1.43)
Bemba	24.88 (4.89-58.40)	66.03 (41.59-95.10)	9.09 (0.01-34.25)	10.85 (0.60)
Lozi	25.72 (11.22-39.53)	64.64 (50.65-85.83)	9.64 (0.01-28.87)	11.45 (0.30)
Tonga	25.65 (18.36-44.91)	62.74 (45.09-72.73)	11.60 (0.01-24.51)	12.74 (0.62)
Ju|'hoansi	100.00 (100.00-100.00)	0.00 (0.00-0.00)	0.00 (0.00-0.00)	100.00 (0.00)
Baka (Cameroon)	0.19 (0.00-2.91)	99.74 (96.22-100.00)	0.07 (0.00-0.87)	95.04 (1.40)
Hadza	17.24 (14.08-20.38)	14.89 (2.01-41.93)	67.87 (42.08-77.65)	83.25 (5.02)
Sabue	7.78 (6.94-8.58)	11.56 (10.01-12.44)	80.65 (79.65-82.31)	99.36 (0.34)
Nzebi	9.42 (4.48-15.46)	86.29 (77.31-93.14)	4.30 (0.01-15.55)	17.46 (0.58)
Nzime	6.85 (0.01-13.69)	78.97 (48.06-88.77)	14.18 (1.65-47.61)	19.45 (0.33)
Khwe	72.45 (46.92-89.56)	17.13 (5.60-52.98)	10.43 (0.01-14.73)	46.84 (3.97)
Amhara	4.14 (2.28-7.24)	0.80 (0.00-5.99)	95.05 (88.26-97.68)	55.56 (0.43)
Zulu	92.49 (84.18-99.99)	7.14 (0.00-13.59)	0.38 (0.00-8.08)	20.73 (0.28)

Table S2 ADMIXTURE components ratio and proportion of KS-, RHG- and eHG-like components in selected populations (%). KS: Khoe-San, RHG: rainforest hunter-gatherer, eHG: eastern African hunter-gatherer. The component ratios correspond to the genetic membership in the KS, RHG and eHG-like clusters respectively, divided by the sum of the three memberships. For each population, the mean ratio as well as the minimum and maximum is given. For the sum, the mean and the standard error of mean is given.

Source 1	Source 2	Target	f_3 statistics	Standard error	Z-score	n SNPs
San ancestry						
Ju|'hoansi (present-day)	Yoruba	BaTwa (Bangweulu)	0.007214	0.000241	29.893	324563
Ju|'hoansi (present-day)	Yoruba	BaTwa (Kafue)	-0.001653	0.000212	-7.802	324637
Ju|'hoansi (present-day)	Yoruba	Bemba	0.019402	0.000269	72.189	322417
Ju|'hoansi (present-day)	Yoruba	Lozi	0.010505	0.000221	47.5	324653
Ju|'hoansi (present-day)	Yoruba	Tonga	0.029379	0.000348	84.427	321758
South Africa LSA[1] 2000 BP	Yoruba	BaTwa (Bangweulu)	0.006406	0.00044	14.555	151367
South Africa LSA[1] 2000 BP	Yoruba	BaTwa (Kafue)	-0.003798	0.000444	-8.553	151514
South Africa LSA[1] 2000 BP	Yoruba	Bemba	0.01885	0.00052	36.225	149807
South Africa LSA[1] 2000 BP	Yoruba	Lozi	0.009628	0.000427	22.561	151212
South Africa LSA[1] 2000 BP	Yoruba	Tonga	0.029431	0.000669	43.978	149358
Rainforest hunter-gatherer ancestry						
Baka (Cameroon, present-day)	Yoruba	BaTwa (Bangweulu)	0.01239	0.000169	73.266	324485
Baka (Cameroon, present-day)	Yoruba	BaTwa (Kafue)	0.009279	0.000146	63.345	324676
Baka (Cameroon, present-day)	Yoruba	Bemba	0.020577	0.000206	100.09	322687
Baka (Cameroon, present-day)	Yoruba	Lozi	0.01219	0.000164	74.113	324731
Baka (Cameroon, present-day)	Yoruba	Tonga	0.031284	0.000285	109.945	322240
BaTwa (Uganda, present-day)	Yoruba	BaTwa (Bangweulu)	0.011806	0.000185	63.726	324983
BaTwa (Uganda, present-day)	Yoruba	BaTwa (Kafue)	0.008921	0.000162	55.084	325107
BaTwa (Uganda, present-day)	Yoruba	Bemba	0.020228	0.000217	93.324	322992
BaTwa (Uganda, present-day)	Yoruba	Lozi	0.011633	0.000173	67.204	324985
BaTwa (Uganda, present-day)	Yoruba	Tonga	0.030853	0.000283	109.16	322444
Shum Laka (3000 BP)	Yoruba	BaTwa (Bangweulu)	0.011391	0.000396	28.758	150207
Shum Laka (3000 BP)	Yoruba	BaTwa (Kafue)	0.007581	0.000388	19.535	150355
Shum Laka (3000 BP)	Yoruba	Bemba	0.019905	0.00049	40.595	148699
Shum Laka (3000 BP)	Yoruba	Lozi	0.011825	0.000389	30.436	150054
Shum Laka (3000 BP)	Yoruba	Tonga	0.02984	0.000593	50.339	148263
Shum Laka (8000 BP)	Yoruba	BaTwa (Bangweulu)	0.012109	0.000398	30.452	149802
Shum Laka (8000 BP)	Yoruba	BaTwa (Kafue)	0.007934	0.000404	19.649	149981
Shum Laka (8000 BP)	Yoruba	Bemba	0.01988	0.000535	37.147	148279
Shum Laka (8000 BP)	Yoruba	Lozi	0.01156	0.000413	27.961	149663
Shum Laka (8000 BP)	Yoruba	Tonga	0.030686	0.000641	47.887	147868
Eastern African hunter-gatherer ancestry						
Hadza (present-day)	Yoruba	BaTwa (Bangweulu)	0.012621	0.000184	68.486	327141
Hadza (present-day)	Yoruba	BaTwa (Kafue)	0.010626	0.00016	66.24	327326
Hadza (present-day)	Yoruba	Bemba	0.020778	0.000222	93.411	325616
Hadza (present-day)	Yoruba	Lozi	0.011851	0.000174	68.042	327277
Hadza (present-day)	Yoruba	Tonga	0.031172	0.000291	107.284	325190
Sabue (present-day)	Yoruba	BaTwa (Bangweulu)	0.013879	0.000204	67.921	324487
Sabue (present-day)	Yoruba	BaTwa (Kafue)	0.012367	0.000198	62.439	324685
Sabue (present-day)	Yoruba	Bemba	0.022015	0.000267	82.545	322229
Sabue (present-day)	Yoruba	Lozi	0.01283	0.000199	64.485	324470
Sabue (present-day)	Yoruba	Tonga	0.032746	0.000332	98.577	321593
Malawi Fingira (2500BP)	Yoruba	BaTwa (Bangweulu)	0.00702	0.000553	12.689	95612
Malawi Fingira (2500BP)	Yoruba	BaTwa (Kafue)	0.000978	0.00054	1.813	95731
Malawi Fingira (2500BP)	Yoruba	Bemba	0.019314	0.000694	27.826	94566
Malawi Fingira (2500BP)	Yoruba	Lozi	0.010178	0.000526	19.351	95537
Malawi Fingira (2500BP)	Yoruba	Tonga	0.029304	0.0008	36.617	94345
Malawi Fingira (6000BP)	Yoruba	BaTwa (Bangweulu)	0.006823	0.001316	5.183	14877
Malawi Fingira (6000BP)	Yoruba	BaTwa (Kafue)	0.00245	0.001231	1.991	14891
Malawi Fingira (6000BP)	Yoruba	Bemba	0.020839	0.001592	13.086	14709
Malawi Fingira (6000BP)	Yoruba	Lozi	0.012779	0.00143	8.938	14878
Malawi Fingira (6000BP)	Yoruba	Tonga	0.030881	0.002059	14.999	14658
Malawi Hora (Holocene)	Yoruba	BaTwa (Bangweulu)	0.008775	0.000543	16.153	88920
Malawi Hora (Holocene)	Yoruba	BaTwa (Kafue)	0.003133	0.00056	5.599	89038
Malawi Hora (Holocene)	Yoruba	Bemba	0.019887	0.000707	28.121	87963
Malawi Hora (Holocene)	Yoruba	Lozi	0.010501	0.000579	18.15	88832
Malawi Hora (Holocene)	Yoruba	Tonga	0.031395	0.000828	37.905	87690

Table S3 f_3 statistics.

[1]Later Stone Age

Admixture source: San (Ju|'hoansi)			
Target	Alpha	Standard error	Z-score
BaTwa (Bangweulu)	0.097506	0.006499	15.003
BaTwa (Kafue)	0.211610	0.006048	34.987
Bemba	0.024346	0.007026	3.465
Lozi	0.002983	0.006406	0.466
Tonga	0.042167	0.007421	5.682
Ba.Kiga	-0.147717	0.007579	-19.489
Nzebi	0.088559	0.006059	14.617
Nzime	0.069584	0.005554	12.528
Southeastern Bantu-speakers	0.203605	0.006959	29.256
Sotho	0.203143	0.005939	34.204
Herero	0.096930	0.008528	11.366
Zulu	0.096930	0.006395	26.572
Igbo	0.017345	0.004932	3.517
Luhya	-0.078049	0.006429	-12.140
Mandinka	-0.060610	0.005938	-10.208
Admixture source: western RHG (Baka)			
Target	Alpha	Standard error	Z-score
BaTwa (Bangweulu)	0.196775	0.012802	15.371
BaTwa (Kafue)	0.427003	0.012378	34.496
Bemba	0.049158	0.014071	3.494
Lozi	0.006057	0.012908	0.469
Tonga	0.085118	0.014858	5.729
Ba.Kiga	-0.298031	0.016229	-18.364
Nzebi	0.178725	0.011910	15.006
Nzime	0.140453	0.010679	13.152
Southeastern Bantu-speakers	0.410843	0.014251	28.828
Sotho	0.409906	0.012499	32.794
Herero	0.195622	0.016904	11.573
Zulu	0.342893	0.012624	27.162
Igbo	0.035037	0.009815	3.570
Luhya	-0.157447	0.013544	-11.624
Mandinka	-0.122275	0.012280	-9.957
Admixture source: eHG (Hadza)			
Target	Alpha	Standard error	Z-score
BaTwa (Bangweulu)	-0.598941	0.069594	-8.606
BaTwa (Kafue)	-1.301033	0.115038	-11.310
Bemba	-0.149053	0.047298	-3.151
Lozi	-0.017539	0.040003	-0.438
Tonga	-0.258693	0.053645	-4.822
Ba.Kiga	0.910673	0.058059	15.685
Nzebi	-0.544107	0.062320	-8.731
Nzime	-0.427371	0.053246	-8.026
Southeastern Bantu-speakers	-1.252155	0.108367	-11.555
Sotho	-1.249356	0.106169	-11.768
Herero	-0.595510	0.076287	-7.806
Zulu	-1.044830	0.093915	-11.125
Igbo	-0.106126	0.033387	-3.179
Luhya	0.481561	0.038566	12.487
Mandinka	0.373773	0.037633	9.932

Table S4 f_4 ratio. Tests how much the target is like the admixture source. The number of SNPs is 337,051 for all tests.

Admixture source: eastern RHG (BaTwa)			
Target	Alpha	Standard error	Z-score
BaTwa (Bangweulu)	0.319966	0.020955	15.269
BaTwa (Kafue)	0.694257	0.023388	29.684
Bemba	0.080020	0.022586	3.543
Lozi	0.009934	0.020960	0.474
Tonga	0.138482	0.023761	5.828
Ba.Kiga	-0.484299	0.032046	-15.113
Nzebi	0.290610	0.019666	14.777
Nzime	0.228385	0.017389	13.134
Southeastern Bantu-speakers	0.667939	0.027276	24.488
Sotho	0.666404	0.024812	26.858
Herero	0.318084	0.027831	11.429
Zulu	0.557486	0.023154	24.077
Igbo	0.057007	0.015852	3.596
Luhya	-0.255826	0.024367	-10.499
Mandinka	-0.198653	0.021705	-9.152
Admixture source: eastern HG (Sabue)			
Target	Alpha	Standard error	Z-score
BaTwa (Bangweulu)	-0.414899	0.039813	-10.421
BaTwa (Kafue)	-0.900871	0.060727	-14.835
Bemba	-0.103373	0.031543	-3.277
Lozi	-0.012337	0.027526	-0.448
Tonga	-0.179299	0.034682	-5.170
Ba.Kiga	0.629962	0.032780	19.218
Nzebi	-0.376881	0.036009	-10.466
Nzime	-0.296019	0.031919	-9.274
Southeastern Bantu-speakers	-0.866789	0.060427	-14.344
Sotho	-0.864923	0.056757	-15.239
Herero	-0.412449	0.046076	-8.951
Zulu	-0.723314	0.052806	-13.698
Igbo	-0.073619	0.022278	-3.305
Luhya	0.333007	0.025598	13.009
Mandinka	0.258576	0.024260	10.659

Table S5 f_4 ratio for additional populations. Tests how much the target is like the admixture source. The number of SNPs is 337,051 for all tests.

Target population	Two-way	Minimum	Maximum	Two-way Ju|'hoansi-Yoruba
BaTwa (Bangweulu)	37.8	33.8	40.9	-
BaTwa (Kafue)	15	13.7	16.9	-
Bemba	22.6	12.5	53.9	-
Lozi	57.2	42.4	66	-
Tonga	41.5	29.6	60.7	-
Baka (Cameroon)	15.5	12.8	18.2	-
Nzime	46.2	37.8	58.4	-
!Xun	17.5	15.3	19.7	21.4
#Khomani	6.5	5.8	7.4	12.7
Karretjie	5.2	4	7	7.2
Southeastern Bantu-speakers	26.2	23.7	28.7	26.3

Table S6 Estimated admixture time in a two-way admixture scenario in MOSAIC [9]. The minimum and maximum values are obtained by bootstrapping individuals. For four populations, the scenario was tested with and without specifying the sources.

BaTwa (Bangweulu)		BaTwa (Kafue)		Bemba		Lozi		Tonga	
Khwe	0.1209	#Khomani	0.0616	Oromo	0.0197	Maasai	0.0236	Maasai	0.0172
Kafue	0.1249	!Xun	0.0656	Amhara	0.0218	Oromo	0.0303	Oromo	0.0204
Maasai	0.1270	Karretjie	0.0677	Maasai	0.0305	Toubou	0.0320	Amhara	0.0246
Sotho	0.1273	Nama	0.0701	Somali	0.0380	BaKiga	0.0324	Somali	0.0323
#Khomani	0.1285	Ju|'hoansi	0.0738	Toubou	0.0438	Amhara	0.0343	Toubou	0.0327

Table S7 F_{ST} estimates between minor ancestry and the closest five panels in the five Zambian populations, two-way admixture scenario with MOSAIC [9].

Marker	Page18	B-M112	E-M5389	E-Z1107	E-U174	E-U209*	E-U290	E-M85	Total
ISOGG	B2a1a1a1∼	B2b	E	E1b1a1∼	E1b1a1a1a1c1a1a	E1b1a1a1a2a1∼	E1b1a1a1a2a1a3b1a	E2b1a	
BaTwa (Bangweulu)	0	1	0	0	8	9	1	2	21
BaTwa (Kafue)	0	1	0	0	3	4	4	0	12
Bemba	1	0	1	11	0	0	0	0	13
Lozi*	3	0	0	15	0	0	0	0	18
Tonga	0	0	0	10	0	0	0	0	10
Total	4	2	1	36	11	13	5	2	74

Table S8 Y chromosome haplogroup counts in the five Zambian populations. *: The following three haplogroups were found in one copy in the Lozi sample and are not shown in the table: I-L801, K-P128,P131,P132, and R-Z93.

Haplogroup	Haplogroup (alt.)	Lozi	Bemba	Tonga	Bisa	Kunda	Luyana	Mbunda
A		0	0	0	0	1	0	0
B		7	1	3	1	0	3	1
E1b1a	E-M2	9	1	2	4	2	5	3
E1b1a7	E-M191	0	0	0	0	1	0	0
E1b1a7a	E-P252/U174	16	4	9	14	12	9	9
E1b1a8	E-U175	54	4	16	16	17	42	31
E1b1b1		0	0	0	0	0	1	0
E2	E-M75	7	0	0	1	2	0	3
R		0	0	0	0	0	1	2
R1		0	0	0	0	1	0	0
R1a	R-SRY10931	1	0	0	0	0	0	0
Total		94	10	30	36	36	61	49

Table S9 Haplogroup counts in a subset of populations from [10]. The nomenclature was updated to fit this paper.

Haplogroup	Haplogroup (ISOGG)	Fwe	Shanjo	Subiya	Totela	Tonga	Mbukushu	Kwamashi
B-M152	B2a1a1a1				0.15	0.03		
B-M112	B2b			0.09	0.08	0.03		
E-M2	E1b1a	0.04	0.08	0	0.23	0.06	0.09	0.08
E-U174	E1b1a1a1a1c1a1a	0.19	0.31	0.18	0.08	0.38	0.18	0.15
E-U175	E1b1a1a1a2a	0.73	0.62	0.73	0.46	0.47	0.55	0.65
E-M75	E2	0.04					0.18	0.12
R	R					0.03		

Table S10 Y haplogroup frequencies in the populations from [11]. The nomenclature was updated to fit this paper.

Population	s^f_{wafr}/s^m_{wafr}	H^X_{wafr}	H^A_{wafr}	s^f_{hg}/s^m_{hg}	H^X_{hg}	H^A_{hg}
BaTwa (Bangweulu)	0.70	0.859	0.914	-3.27	0.141	0.086
BaTwa (Kafue)	0.58	0.748	0.821	-10.04	0.252	0.179
Bemba	0.79	0.928	0.966	-1.83	0.072	0.034
Lozi	0.83	0.927	0.957	-2.78	0.073	0.043
Tonga	0.82	0.926	0.958	-2.53	0.074	0.042
Baka (Cameroon)	0.36	0.599	0.710	-14.01	0.401	0.290
Baka (Gabon)	0.24	0.576	0.723	-4.41	0.424	0.277
Bongo (east)	0.44	0.722	0.829	-3.23	0.278	0.171
Bongo (south)	0.30	0.646	0.786	-3.06	0.354	0.214
Batwa	0.52	0.645	0.720	9.16	0.355	0.280
Khutse San	1.99	0.222	0.200	0.85	0.778	0.800
Karretjie	0.10	0.118	0.163	1.38	0.882	0.837
#Khomani	1.69	0.155	0.143	0.92	0.845	0.857
Khwe	0.80	0.588	0.610	1.41	0.412	0.390
Nama	-0.18	0.078	0.150	1.68	0.922	0.850
!Xun	0.36	0.170	0.201	1.27	0.830	0.799
Maasai	1.78	0.964	0.881	-0.35	0.036	0.119
Luhya	1.00	0.953	0.953	0.94	0.047	0.047
Nzebi	0.82	0.920	0.951	-3.08	0.080	0.049
Nzime	0.84	0.926	0.953	-3.75	0.074	0.047
Ba.Kiga	1.00	0.946	0.946	1.05	0.054	0.054
Southeastern Bantu-speakers	0.76	0.772	0.808	3.61	0.228	0.192
Herero	0.59	0.834	0.913	-2.16	0.166	0.087
Ju|'hoansi (source)	1.00	0.000	0.000	1.00	1.000	1.000
Yoruba (source)	1.00	1.000	1.000	1.00	0.000	0.000

Table S11 Ratios of female to male ancestry contributions for the western-African source (Yoruba, s^f_{wafr}/s^m_{wafr}) and the hunter-gatherer source (Ju|'hoansi, s^f_{hg}/s^m_{hg}), and mean ancestry proportions for the X chromosome and autosomes (H^X_{wafr} and H^A_{wafr}: western-African source, H^X_{hg} and H^A_{hg}: hunter-gatherer source). Negative ratios indicate model failure, for example because the sex-biased admixture happened over more than one generation.

Population	H^X_{wafr}/H^A_{wafr}	H^X_{wafr}	H^A_{wafr}	H^X_{hg}/H^A_{hg}	H^X_{hg}	H^A_{hg}
BaTwa (Bangweulu)	0.94	0.859	0.914	1.63	0.141	0.086
BaTwa (Kafue)	0.91	0.748	0.821	1.41	0.252	0.179
Bemba	0.96	0.928	0.966	2.13	0.072	0.034
Lozi	0.97	0.927	0.957	1.71	0.073	0.043
Tonga	0.97	0.926	0.958	1.77	0.074	0.042
Baka (Cameroon)	0.84	0.599	0.710	1.38	0.401	0.290
Baka (Gabon)	0.80	0.576	0.723	1.53	0.424	0.277
Bongo (east)	0.87	0.722	0.829	1.63	0.278	0.171
Bongo (south)	0.82	0.646	0.786	1.66	0.354	0.214
Batwa	0.90	0.645	0.720	1.27	0.355	0.280
Khutse San	1.11	0.222	0.200	0.97	0.778	0.800
Karretjie	0.73	0.118	0.163	1.05	0.882	0.837
Khomani	1.09	0.155	0.143	0.99	0.845	0.857
Khwe	0.96	0.588	0.610	1.06	0.412	0.390
Nama	0.52	0.078	0.150	1.08	0.922	0.850
Xun	0.84	0.170	0.201	1.04	0.830	0.799
Maasai	1.09	0.964	0.881	0.30	0.036	0.119
Luhya	1.00	0.953	0.953	0.99	0.047	0.047
Nzebi	0.97	0.920	0.951	1.65	0.080	0.049
Nzime	0.97	0.926	0.953	1.58	0.074	0.047
Bakiga	1.00	0.946	0.946	1.01	0.054	0.054
Southeastern Bantu-speakers	0.96	0.772	0.808	1.19	0.228	0.192
Herero	0.91	0.834	0.913	1.91	0.166	0.087
Ju—'hoansi (source)	1.00	0.000	0.000	1.00	1.000	1.000
Yoruba (source)	1.00	1.000	1.000	1.00	0.000	0.000

Table S12 X chromosomal to autosomal ancestry ratio and mean ancestry proportions for the autosomes and the X chromosome (H^A and H^X, as in Supplementary Table S11) for the western-African source (Yoruba) and the hunter-gatherer source (Ju|'hoansi). A ratio greater than 1 indicates a female biased contribution, while a ratio less than 1 indicates a male biased contribution.

Population	n[1]	n_{final}[2]	Dataset	Group	Country of origin	Individual ID
Kenya IA[3] Deloraine	1	1	[12]	WA[6] ancestry agriculturalists	Kenya	
Tanzania Pemba 700 BP	1	1	[13]	WA[6] ancestry agriculturalists	Tanzania	
Kenya Pastoral IA[3]	3	3	[12]	EA[7] Pastoralists	Kenya	
Kenya Pastoral Neolithic Elmenteitan	5	5	[12]	EA[7] Pastoralists	Kenya	
Kenya Pastoral Neolithic	11	10	[12]	EA[7] Pastoralists	Kenya	
Tanzania Luxmanda 3000 BP	1	1	[13]	EA[7] Pastoralists	Tanzania	
Tanzania PN[4]	4	4	[13]	EA[7] Pastoralists	Tanzania	
Mota	1	1	[14]	eHG[8]	Ethiopia	
Kenya 500 BP	1	1	[12]	eHG[8]	Kenya	
Kenya LSA[5]	1	1	[12]	eHG[8]	Kenya	
Tanzania Zanzibar 1400 BP	1	1	[13]	eHG[8]	Tanzania	
Tanzania Pemba 1400 BP	1	1	[13]	eHG[8]	Tanzania	
Malawi Fingira 2500 BP	1	1	[13]	eHG[8]	Malawi	I4426
Malawi Fingira 6000 BP	1	1	[13]	eHG[8]	Malawi	I4427
Malawi Hora Holocene	1	1	[13]	eHG[8]	Malawi	I2966
Shum Laka 3000 BP	2	2	[15]	RHG	Cameroon	4/A, 5/B
Shum Laka 8000 BP	2	2	[15]	RHG	Cameroon	2/SEI, 2/SEII
SA[10] IA[3] 500 BP	4	4	[16]	WA[6] ancestry agriculturalists	SA[10]	cha001, ela001, mfo001, new001
SA[10] LSA[5] Pastoralist 1200 BP	1	1	[13]	Pastoralists	SA[10]	I9134
SA[10] LSA[5] Cape 2000 BP	2	2	[13]	KS[9]	SA[10]	I9133, I9028
Ballito Bay	2	2	[16]	KS[9]	SA[10]	baa001, bab001
Total	47	46				

Table S13 Information about the ancient DNA samples.

[1]Sample size.

[2]Sample size retained for the analyses.

[3]Iron Age.

[4]Pastoral Neolithic.

[5]Later Stone Age.

[6]Western African.

[7]Eastern African.

[8]Eastern African hunter-gatherer.

[9]Khoe-San.

[10]South Africa.

Defining marker(s)	Haplogroup (SNAPPY)	Haplogroup (ISOGG 2019 v15.58)	rs number	Position (hg37)
Page18	B2a	B2a1a1a1~		
B-M112	B2b	B2b		
E-M96/PF1823[1]	E	E		
E-Z1107	E1b1a1	E1b1a1~		
E-P252/U174	E1b1a1a1c1a1	E1b1a1a1a1c1a1a	rs16980586	Y:16251357
E-P277,P278.1*	E1b1a1a1d1	E1b1a1a1a2a		
E-U175		E1b1a1a1a2a	rs16980588	Y:16253694
E-U209*	E1b1a1a1d1	E1b1a1a1a2a1~	rs16980406	Y:21646058
E-U290	E1b1a1a1d1a	E1b1a1a1a2a1a3b1a	rs16980502	Y:17294958
E-M85	E2b1a	E2b1a		
I-L801	I2a2a1c2a	I2a1b1a2b1		
K-M9*	K	K		
K-P128,P131,P132*	K	K~		
R-Z93	R1a1a1b2	R1a1a1b1a1a1c~		

Table S14 Different names for the same haplogroup. [1]The defining marker in SNAPPY is M5389. *These two markers (P277,P278.1,U209 and M9,P128,P131,P132) correspond to a single haplogroup in SNAPPY (E1b1a1a1d1 and K) and to two different haplogroups in ISOGG.

Supplementary Notes

Y chromosome haplogroups

A total of eleven different Y-chromosome haplogroups were identified with SNAPPY [17] among the 74 males from Zambia, with an accuracy score varying from 0.786 to 1 (for one and 70 haplogroups respectively). The most represented major haplogroup was E (68 individuals, 88%), and in particular the haplogroup E-M2 sub-lineages group (84%). E-M2 (defined by the marker M291 and also referred to as E1b1a1) is a common haplogroup in Bantu-speaking populations [10, 18] and its present-day distribution is associated with the Bantu expansion [18]. The next most represented haplogroup was haplogroup B (six individuals, 0.8%). Haplogroups I (I-L801), K (K-P128,P131,P132), and R (R-Z93), common among non-Africans, were found in one individual each in the Lozi population (Supplementary Table S8). The individuals with the haplogroups I and R also showed recent European admixture in autosomal analyses.

Regarding haplogroup E more specifically, we found several copies of haplogroups E1b1a1a1a1c1a1a (E-U174), E1b1a1a1a2a1 (E-U209), and E1b1a1a1a2a1a3b1a (E-U290) in the Zambian BaTwa populations. These haplogroups have a pan-African distribution [19]. In a study of diverse African populations [18], high frequencies of E-U174 and E-U290 were found in Niger-Congo speaking populations such as the Yoruba and several populations from central Africa, in particular from southern Cameroon (E-U174 is present at 51.92% in the Bamileke and E-U290 at 61.29% in the Ewondo).

We were able to compare our results to previous results from Zambian populations. Y-haplogroups were reported for a total of 549 Zambian individuals [10] (including the populations from the Luangwa valley first reported in [20]) using 31 biallelic markers and 12 microsatellites. We selected a subset of groups (Lozi, Bemba, Tonga, Bisa. Kunda, Luyana and Mbunda), because i) we have samples from the same populations; ii) the samples come from the Luangwa valley; or iii) the sample size was large. We summarized their results using the same nomenclature like is used today in Supplementary Table S9. Two haplogroups were particularly frequent: E-U174 (or E1b1a7a; 15-40%) and E-U175 (E1b1a8; 40-79%). Thus, E-U174, which is common in the BaTwa populations, is also common in other Zambian populations. E-U209 (E1b1a1a1a2a1∼), another haplogroup common in the Zambian BaTwa populations, was not typed in [10], but it derives from E-U175 (E1b1a1a1a2a) which was typed; we can hypothesize that the E-U175 haplogroups are in fact E-U209. Similar results were found in another study of Y chromosome diversity in Zambian populations [11] (summarized with updated nomenclature in Supplementary Table S10).

Additionally, two individuals with E2b1a (E-M85) were found in the BaTwa population from Bangweulu. Haplogroup E2 has also previously been reported in Zambian populations [10, 11].

In our sample of Zambian agropastoralists, the majority of haplogroups E is represented by E1b1a1 (x E1b1a1a1a1c1a1a, E1b1a1a1a2a1, E1b1a1a1a2a1a3b1a) (E-Z1107). This contrasts the result in the BaTwa and previous results [10, 11] and is likely a technical artefact: the defining markers U174, U175 and U290 showed high levels of missingness (19-26%) in the three agropastoralist populations. Moreover, the total genotyping rate for the Y chromosome for the agropastoralists was lower than

for the BaTwa (0.9457 versus 0.9890). Further investigation of the samples is required to confirm this.

We found two haplogroups B, B2a1a1a1∼ (B-Page18; one copy in the Bemba and three copies in the Lozi) and B2b (B-M112; one copy in each BaTwa population). Haplogroup B2a is associated with Bantu-speakers [21, 22], while haplogroup B2b is associated with African hunter-gatherer populations [22, 23]. These differences in the haplogroup B sub-lineages, though based on few haplotypes, suggest a difference in ancestry between the agropastoralist and the BaTwa communities. Haplogroup B lineages were previously reported for Zambian populations. The frequency was 10%, 7% and 10% respectively in the Bemba, Lozi and Tonga [10]. And copies of B2a1a1a1 and B2b were found in Tonga, Subiya and Totela populations sample (in one or two copies in samples of 11 to 32 individuals) [11](Supplementary Table S10).

Testing the origin of the RHG-like component in the Zambian populations

The introduction of the RHG-like component in the BaTwa could be associated with the introduction of western-African-like (Bantu-speaker) ancestry, through the arrival into Zambia of a group that carried both ancestries. We tested this hypothesis with analyses where we specified reference populations, which should enable to characterize two-step-admixture histories; and by comparing genetic cluster memberships proportions results to qualitative expectations under a simple scenario.

We specified reference populations in MOSAIC. MOSAIC should be able to characterize admixture histories such as: population a admixed with population b, and later the resulting population $a+b$ admixed with population c. We hypothesized that this analysis could estimate the time of the admixture between a RHG-like population and a farmer population, if such an event predated the admixture with a KS-like population. We tested a three-way admixture scenario with the Yoruba, Baka (RHG from Cameroon) and Ju|'hoansi (KS); and a four-way admixture scenario in which we added the Amhara agriculturalists from eastern Africa. We observed timings similar to those reported earlier, and consistent across the different co-ancestry curves, particularly for the three-way scenario (Supplementary Figures S20, S21), which favors a single admixture event (note though that the dates are younger than in the two-way scenario for the BaTwa from Bangweulu, closer to 25 than to 38 generations ago). The hypothesis of a single admixture event is reinforced with the observation that the minor ancestries co-localize in the karyograms. We also note that some of the co-ancestry curves in the four-way scenario do not have the expected shape, suggesting that this model does not fit the data well (Supplementary Figures S22, S23). Three- and four-way admixture scenarios without specifying a reference panel gave similar results (see plots in the GitHub repository [24]), and the karyograms showed a colocalization of the minor ancestries.

We also investigated the question of the origin of the RHG-like component with the genetic membership proportions obtained with the unsupervised clustering approach. The Zambian agropastoralists are a good proxy for the major admixture source in the BaTwa and they have some RHG-like component that was likely introduced during the Bantu expansion. We wanted to test whether the RHG-like component in the BaTwa

could be entirely due to the major source population (that was already admixed compared to e.g. the Yoruba). If the hypothesis is correct, and if we assume that there was no selection against the RHG-like component in the Zambian agropastoralists, we expect that the ratio RHG/western-African-like component would be similar in the BaTwa and in the Zambian agropastoralists. This is not the case (Figure S19); the mean ratio is significantly greater in the two BaTwa populations (Welch two sample t-test, BaTwa from Kafue versus Lozi: p-value=$1.5*10^{-14}$, BaTwa from Bangweulu versus Lozi: p-value=$6.3*10^{-7}$). The three agropastoralist populations have the same mean (non significant p-values for the three pairwise comparisons). We note that the BaTwa from Kafue have a significantly greater mean than the BaTwa from Bangweulu (p-value=$3.8*10^{-10}$).

These results suggest that the RHG-like component in the BaTwa cannot be solely explained by its presence in the major source. It must, at least partially, have a different origin; for example the population that contributed the minor source to the BaTwa could have some genetic connection to RHG (despite being predominantly KS-like). Another alternative would be that there has been selection against the RHG-like variants in the Zambian agropastoralists, resulting in lower fractions than in the BaTwa; however such a pattern is unlikely, particularly at a genome-wide scale.

Supplementary Discussion

Admixture time estimates: comparison between MOSAIC and previous studies

We compared the admixture time estimates inferred by MOSAIC [9] to estimates from a LD decay-based method [25] for the southeastern Bantu-speakers from South Africa. For two-way admixture, we obtained an estimate of ∼26 generations ago with MOSAIC (Supplementary Table S6); the LD based estimate is slightly older at ∼31 generations ago [26] but overall this confirms that our admixture time estimates are consistent with previous methods and results.

References

[1] Behr, A. A., Liu, K. Z., Liu-Fang, G., Nakka, P. & Ramachandran, S. pong: fast analysis and visualization of latent clusters in population genetic data. *Bioinformatics* **32**, 2817–2823 (2016).

[2] Gurdasani, D. *et al.* The African Genome Variation Project shapes medical genetics in Africa. *Nature* **517**, 327–332 (2015).

[3] Patin, E. *et al.* The impact of agricultural emergence on the genetic history of African rainforest hunter-gatherers and agriculturalists. *Nature Communications* **5**, 3163 (2014).

[4] Fortes-Lima, C. A. *et al.* The genetic legacy of the expansion of Bantu-speaking peoples in Africa. *Nature* **625**, 540–547 (2024).

[5] Auton, A. *et al.* A global reference for human genetic variation. *Nature* **526**, 68–74 (2015).

[6] Scheinfeldt, L. B. *et al.* Genomic evidence for shared common ancestry of East African hunting-gathering populations and insights into local adaptation. *Proceedings of the National Academy of Sciences of the United States of America* **116**, 4166–4175 (2019).

[7] Schlebusch, C. M. *et al.* Genomic variation in seven Khoe-San groups reveals adaptation and complex African history. *Science* **338**, 374–379 (2012).

[8] Haber, M. *et al.* Chad Genetic Diversity Reveals an African History Marked by Multiple Holocene Eurasian Migrations. *The American Journal of Human Genetics* **99**, 1316–1324 (2016).

[9] Salter-Townshend, M. & Myers, S. Fine-scale inference of ancestry segments without prior knowledge of admixing groups. *Genetics* **212**, 869–889 (2019).

[10] de Filippo, C. *et al.* Y-chromosomal variation in sub-Saharan Africa: insights into the history of Niger-Congo groups. *Molecular Biology and Evolution* **28**, 1255–1269 (2011).

[11] Barbieri, C., Butthof, A., Bostoen, K. & Pakendorf, B. Genetic perspectives on the origin of clicks in Bantu languages from southwestern Zambia. *European Journal of Human Genetics* **21**, 430–436 (2013).

[12] Prendergast, M. E. *et al.* Ancient dna reveals a multistep spread of the first herders into sub-Saharan Africa. *Science* **365** (2019).

[13] Skoglund, P. *et al.* Reconstructing Prehistoric African Population Structure. *Cell* **171**, 59–71.e21 (2017).

[14] Gallego Llorente, M. *et al.* Ancient Ethiopian genome reveals extensive Eurasian admixture in Eastern Africa. *Science* **350**, 820–822 (2015).

[15] Lipson, M. *et al.* Ancient West African foragers in the context of African population history. *Nature* **577**, 665–670 (2020).

[16] Schlebusch, C. M. *et al.* Southern African ancient genomes estimate modern human divergence to 350,000 to 260,000 years ago. *Science* **358**, 652–655 (2017).

[17] Severson, A. L. *et al.* SNAPPY: Single Nucleotide Assignment of Phylogenetic Parameters on the Y chromosome. *bioRxiv* 454736 (2018).

[18] D'Atanasio, E. *et al.* The peopling of the last Green Sahara revealed by high-coverage resequencing of trans-Saharan patrilineages. *Genome Biology* **19**, 20 (2018).

[19] Larmuseau, M. H. D. *et al.* The Paternal Landscape along the Bight of Benin – Testing Regional Representativeness of West-African Population Samples Using Y-Chromosomal Markers. *PLOS One* **10**, e0141510 (2015).

[20] de Filippo, C., Heyn, P., Barham, L., Stoneking, M. & Pakendorf, B. Genetic perspectives on forager-farmer interaction in the Luangwa valley of Zambia. *American Journal of Physical Anthropology: The Official Publication of the American Association of Physical Anthropologists* **141**, 382–394 (2010).

[21] Naidoo, T. *et al.* Development of a single base extension method to resolve Y chromosome haplogroups in sub-Saharan African populations. *Investigative Genetics* **1** (2010).

[22] Barbieri, C. *et al.* Refining the Y chromosome phylogeny with southern African sequences. *Human Genetics* **135**, 541–53 (2016).

[23] Naidoo, T. *et al.* Y-Chromosome Variation in Southern African Khoe-San Populations Based on Whole-Genome Sequences. *Genome Biology and Evolution* **12**, 1031–1039 (2020).

[24] Breton, G. zambia_batwa (2024). GitHub doi 10.5281/zenodo.11623128.

[25] Patterson, N. *et al.* Ancient admixture in human history. *Genetics* **192**, 1065–93 (2012).

[26] Schlebusch, C. M., Prins, F., Lombard, M., Jakobsson, M. & Soodyall, H. The disappearing San of southeastern Africa and their genetic affinities. *Human Genetics* **135**, 1365–1373 (2016).
