# Supplementary material for: BaTwa populations from Zambia retain ancestry of past hunter-gatherer groups
Source: Nat Commun. 2024 Aug 24;15:7307. doi: 10.1038/s41467-024-50733-y (PMC11344834; doi:10.1038/s41467-024-50733-y)
Supplement: Supplementary file 3 — Reporting Summary [file 41467_2024_50733_MOESM3_ESM.pdf]

nature portfolio

Corresponding author(s): Mattias Jakobsson

Last updated by author(s): Jun 11, 2024

Reporting Summary

Nature Portfolio wishes to improve the reproducibility of the work that we publish. This form provides structure for consistency and transparency in reporting. For further information on Nature Portfolio policies, see our Editorial Policies and the Editorial Policy Checklist.

Statistics

For all statistical analyses, confirm that the following items are present in the figure legend, table legend, main text, or Methods section.

n/a	Confirmed	
☐	☒	The exact sample size (*n*) for each experimental group/condition, given as a discrete number and unit of measurement
☐	☒	A statement on whether measurements were taken from distinct samples or whether the same sample was measured repeatedly
☐	☒	The statistical test(s) used AND whether they are one- or two-sided<br>*Only common tests should be described solely by name; describe more complex techniques in the Methods section.*
☒	☐	A description of all covariates tested
☒	☐	A description of any assumptions or corrections, such as tests of normality and adjustment for multiple comparisons
☐	☒	A full description of the statistical parameters including central tendency (e.g. means) or other basic estimates (e.g. regression coefficient) AND variation (e.g. standard deviation) or associated estimates of uncertainty (e.g. confidence intervals)
☐	☒	For null hypothesis testing, the test statistic (e.g. *F*, *t*, *r*) with confidence intervals, effect sizes, degrees of freedom and *P* value noted<br>*Give P values as exact values whenever suitable.*
☒	☐	For Bayesian analysis, information on the choice of priors and Markov chain Monte Carlo settings
☒	☐	For hierarchical and complex designs, identification of the appropriate level for tests and full reporting of outcomes
☐	☒	Estimates of effect sizes (e.g. Cohen's *d*, Pearson's *r*), indicating how they were calculated

Our web collection on statistics for biologists contains articles on many of the points above.

Software and code

Policy information about availability of computer code

Data collection	BaTwa data: 40 samples for each of two populations, genotyped on the H3Africa Consortium Array v1 (H3Africa_2017_20021485_A2, Ramsay et al. 2016), implemented on the Illumina Infinium array. The results were analysed with the software GenomeStudio 2011.1 from Illumina Inc, and aligned to the human genome hg37.
Data analysis	We used plink v1.90b4.9 (Purcell et al. 2007) for filtering and merging datasets, and for some analysis (such as identifying related individuals and inferring ROH). KING v2.1.4 (Manichaikul et al. 2010) was used for identifying related individuals. asd v1.0 (Szpiech, https://github.com/szpiech/asd) was used to visually inspect the data. The data was phased with SHAPEIT2 v2.r904 (Delaneau et al. 2012) with the 1000 Genomes phase 3 haplotype panel (Auton et al. 2015) and the 1000 Genomes combined map for hg37. eigensoft v7.2.0 smartpca (Patterson et al. 2006) was used for principal component analyses, and plotted with R v3.6.3 (R Team, 2013). ADMIXTURE v1.3.0 (Alexander et al. 2009) was used for unsupervised and supervised genetic clustering of the dataset; the results were plotted with pong v1.4.7 (Behr et al. 2016). We explored the space of admixture graphs with the find_graphs tool of ADMIXTOOLS 2 (Maier and Patterson 2023), as coded in the R package admixtools version 2.0.0. AdmixTools v5.0-20171024 (Patterson et al. 2012) was used to compute various f statistics (qp3Pop and qpF4ratio). MOSAIC v1.3.6 (Salter-Townshend and Myers 2019) was used to characterize admixture events. Y chromosome haplogroups were called with SNAPPY v0.1 (Severson et al. 2018) and Python v2.7.6, and matched with the haplogroup in the ISOGG tree Y-DNA Haplogroup Tree 2019 V15.58 2020.

For manuscripts utilizing custom algorithms or software that are central to the research but not yet described in published literature, software must be made available to editors and reviewers. We strongly encourage code deposition in a community repository (e.g. GitHub). See the Nature Portfolio guidelines for submitting code & software for further information.

Data

Policy information about availability of data

All manuscripts must include a data availability statement. This statement should provide the following information, where applicable:

- Accession codes, unique identifiers, or web links for publicly available datasets
- A description of any restrictions on data availability
- For clinical datasets or third party data, please ensure that the statement adheres to our policy

The genotype data generated in this study for the Zambian BaTwa individuals have been deposited in the European Genome-Phenome Archive (EGA) under accession code EGA50000000364. The genotype data are available under restricted access for non-commercial projects addressing population and ancestry research questions. Access is granted only to the applicant and for a specific project. Access can be obtained by applying to the EGA Data Access Committee EGAC50000000258.
The genotype data for the Bemba, Lozi and Tonga (Fortes Lima et al. 2024) used in this study are available in the EGA database under accession code EGAS50000000006 (https://ega-archive.org/studies/EGAS50000000006).
The genotype data for the Dinka, Hadza and Sabue (Scheinfeldt et al. 2019) used in this study are available in the NIH dbGAP repository under accession code phs001780.v1.p1 (https://www.ncbi.nlm.nih.gov/projects/gap/cgi-bin/study.cgi?study_id=phs001780.v1.p1). The authorized NIH Data Access Committee (DAC) granted data access to CMS (date of approval: 2019-05-17).
The genotype data for the Ba.Kiga, Baka (from Cameroon and Gabon), BaTwa (from Uganda), Bemba, Bongo, Nzebi and Nzime (Patin et al. 2014) used in this study are available in the EGA database under accession code EGAS00001000605 (https://ega-archive.org/studies/EGAS00001000605). Data access was granted by the EGA DAC EGAC00001000139 (date of approval: 2021-05-07).
The genotype data for the Amhara, Igbo, Mandinka, Oromo, Somali, Sotho and Zulu (Gurdasani et al. 2015) used in this study are available in the EGA database under accession code EGAS00001000959 (https://ega-archive.org/studies/EGAS00001000959).
The genotype data for the Sara and Toubou (Haber et al. 2016) used in this study are available in the EGA database under accession code EGAD00010001103 (https://ega-archive.org/datasets/EGAD00010001103) under the standard Sanger publication policy.
The ancient DNA data (Vicente and Schlebusch 2020) used in this study is available upon request.
The map used in Figure 1a is available in the figshare database (https://doi.org/10.6084/m9.figshare.6396959.v1) (Beck et al. 2018 version 1).
The map used in Figure 1b is in the public domain (https://www.naturalearthdata.com).

Research involving human participants, their data, or biological material

Policy information about studies with human participants or human data. See also policy information about sex, gender (identity/presentation), and sexual orientation and race, ethnicity and racism.

Reporting on sex and gender

Participants were asked to identify their sex. The forms, that were approved by ethics committees, did not distinguish between sex and gender.
We did not perform analyses related to gender.
To identify the genotypic sex of the samples for which we did not have that information, we ran plink "--check-sex" without parameters and plotted the resulting X chromosome inbreeding coefficients (F estimates). After visual inspection (in which one can see two clusters, one for females and one for males), we set thresholds and created a new fileset with information about the sex of all individuals with plink "--impute-sex 0.5 0.9". This was used for calculating the X chromosome to autosomes ancestry ratio (as we needed to specify which samples are haploid for the X chromosome).
We calculated female and male contributions for two ancestries of interest.
We identified the Y chromosome haplogroup of the Zambian male samples. This was performed for 33 Zambian BaTwa individuals, and 41 Zambian agropastoralists.

Reporting on race, ethnicity, or other socially relevant groupings

We were aware that some individuals may be reluctant to self-identify and no one was asked to do so directly. We asked instead for their clan affiliation and that of their parents and grandparents from participants whose families have been resident locally over two generations to improve the chances of sampling those with BaTwa ancestry. The term BaTwa was not included in the participant information sheet, but the project aim of looking for the pre-Bantu ancestry of the region was understood as an indirect reference to the BaTwa. Some participants did self-identify as BaTwa (plural of MuTwa, a single individual), a term understood to reflect a shared island lifestyle (fishing and hunting, and more recently with some horticulture and keeping of cattle following restrictions on hunting).

Population characteristics

Population characteristics (size, distribution, age profile) are not available as census data does not record ethnicity. Samples were collected only from adults 18 years old and older.

Recruitment

In the Lake Bangweulu region we worked within Chief Bwaylyaponda's chiefdom and two attendants were allocated to take us to BaTwa villages on two islands/villages where BaTwa headmen assisted in explaining the project to the communities involved. In the Kafue Flats, the BaTwa area straddles two chiefdoms, that of Hamusonde and Choongo (a chief of BaTwa ancestry). Three islands/villages were visited with the most prominent headman a MuTwa (singular for BaTwa). Main guide was an officer from the National Parks and Wildlife Department, familiar with the BaTwa, assisted by local BaTwa individuals. The results of the project were disseminated directly to the island villagers in July 2022 in fullfillment of the University of Zambia ethics requirements.

Ethics oversight

Ethical clearance was granted by the Committee on Research Ethics of the University of Liverpool, UK (permit number RETH001037), as well as the University of Zambia Biomedical Research Ethics Committee (permit number 016-07-15), the National Health Research Authority, Zambia (permit number MH/101/23/10/1) and the Swedish Ethical Review Authority (Dnr 2021-04013). Written informed consent was obtained from the participants before saliva samples were collected. The results were returned to the populations in July 2022, prior to submission to any journal.

Note that full information on the approval of the study protocol must also be provided in the manuscript.

Field-specific reporting

Please select the one below that is the best fit for your research. If you are not sure, read the appropriate sections before making your selection.

[x] Life sciences [ ] Behavioural & social sciences [ ] Ecological, evolutionary & environmental sciences

For a reference copy of the document with all sections, see nature.com/documents/nr-reporting-summary-flat.pdf

Life sciences study design

All studies must disclose on these points even when the disclosure is negative.

Sample size	40 individuals were sampled to represent Batwa from the north and central Zambia (in total 80 individuals). After filtering of related individuals, we retained 36 individuals in one, 33 in the other population. The sample size was a balance between practical challenges of sampling in these remotes regions of Zambia, and the number of individuals needed to draw conclusions about a population.<br>Sample sizes for comparative populations are listed in Supplementary Table 1.
Data exclusions	Samples were excluded in order to not have first and second-degree relatives in the dataset. This is a common procedure, as relatedness impacts several statistical analyses. The comparative populations were downsampled to 36 individuals, in order to have similar sample sizes, as sample size can impact analyses.<br>In some analyses, individual samples that had recent admixture from other populations (specifically, two Lozi individuals with recent non-African admixture and one BaTwa from Uganda with western African admixture) were excluded. This was done to focus on the main genetic background in the population.
Replication	Thousands to millions of genetic markers were analysed as an internal replication of the results. Detailed description of the methods used, including samples included in the dataset, software employed and respective parameters is available in the Methods section. All attempts to repeat the experiment (for example with different densities of variants) were successful.
Randomization	Randomization in not applicable in this study. Individuals are grouped in populations according to sampling. One focus of the study is to test whether these populations have a common genetic background.
Blinding	Blinding is not applicable to this study. The ethnicity of each individual analysed was determining for sampling, as this study aim at characterizing specific groups.

Reporting for specific materials, systems and methods

We require information from authors about some types of materials, experimental systems and methods used in many studies. Here, indicate whether each material, system or method listed is relevant to your study. If you are not sure if a list item applies to your research, read the appropriate section before selecting a response.

Materials & experimental systems

n/a	Involved in the study	
[x]	[ ]	Antibodies
[x]	[ ]	Eukaryotic cell lines
[x]	[ ]	Palaeontology and archaeology
[x]	[ ]	Animals and other organisms
[x]	[ ]	Clinical data
[x]	[ ]	Dual use research of concern
[x]	[ ]	Plants

Methods

n/a	Involved in the study	
[x]	[ ]	ChIP-seq
[x]	[ ]	Flow cytometry
[x]	[ ]	MRI-based neuroimaging
